# A systematic literature review of CVID reveals pervasive detrimental noninfectious manifestations

**DOI:** 10.70962/jhi.20250157

**Published:** 2025-10-24

**Authors:** G. Michelle Ducasa, Rebecca A. Marsh, Ali Mojebi, Hsi-en Ho, Charlotte Cunningham-Rundles, Lisa Forbes Satter, Elena W.Y. Hsieh, Sinisa Savic, Marta Dafne Cabañero-Navalón, Hector Balastegui-Martin, Joud Hajjar, Gulbu Uzel, Kevin S. Thorneloe, Jocelyn R. Farmer

**Affiliations:** 1 Precision AQ, Bethesda, MD, USA; 2 Pharming Healthcare, Inc, Warren, NJ, USA; 3 Icahn School of Medicine at Mount Sinai, New York, NY, USA; 4 Texas Children’s Hospital, Baylor College of Medicine, Houston, TX, USA; 5 University of Colorado Anschutz School of Medicine, Aurora, CO, USA; 6 Leeds Institute of Rheumatic and Musculoskeletal Medicine, University of Leeds, Leeds, UK; 7 National Institute for Health and Care Research Leeds Biomedical Research Centre, Leeds, UK; 8 Primary Immunodeficiencies Unit, Department of Internal Medicine, University and Polytechnic Hospital La Fe, Valencia, Spain; 9 National Institute of Allergy and Infectious Diseases, National Institutes of Health, Bethesda, MD, USA; 10 Lahey Hospital and Medical Center, Beth Israel Lahey Health, Burlington, MA, USA

## Abstract

Noninfectious manifestations of common variable immunodeficiency (CVID) are not formally summarized. We performed a systematic literature review to generate a comprehensive reference for the field. Splenomegaly was the most reported manifestation across 53 publications, occurring in a median of 35.2% of patients. Frequently reported digestive system manifestations included diarrhea (median 27.8%; 21 publications), hepato(spleno)megaly (median, 21.0%; 19 publications), portal hypertension (median 21.0%; 3 publications), nodular lymphoid hyperplasia (median, 17.0%; 9 publications), and enteropathy (median, 16.0%; 34 publications). Other notable manifestations included interstitial lung disease (median, 8.7%; 32 publications) and autoimmune cytopenias (median 18.0%; 21 publications). Steroids and rituximab were the most frequently reported treatments. Numerous manifestations significantly adversely affected survival, including lymphoma, granulomatous lymphocytic interstitial lung disease, splenomegaly, and liver diseases. These comprehensive data document the pervasiveness and negative impact of noninfectious manifestations in CVID and support a call to action to develop novel therapeutics.

## Teaser

Noninfectious manifestations of CVID have not been formally summarized. This systematic literature review provides a comprehensive summary as a reference point for the field and documents the pervasiveness and negative impact of noninfectious manifestations driven by immune dysregulation.

## Introduction

Common variable immunodeficiency (CVID) is a phenotypic inborn error of immunity (IEI) diagnosis given to patients with heterogenous manifestations characterized by defective B cell function and impaired immunoglobulin (Ig) production (1, 2). A CVID diagnosis can encompass diseases with known and unknown monogenic causes as well as complex etiologies such as somatic variants, polygenic disease, and multifactorial mechanisms (3). An ongoing debate is whether monogenic CVID disorders should be referred to as CVID or conceptualized individually based on their genetic driver (4). CVID affects ∼1 in 10,000 to 1 in 100,000 persons and represents the most common symptomatic IEI (5). Consensus definitions vary but generally require the presence of marked hypogammaglobulinemia, including low levels of IgG and either IgA or IgM, reduced capability to mount specific antibody responses, and the exclusion of secondary causes (5, 6, 7). The immune impairment observed in patients with CVID leads to recurrent infections, particularly of the sinopulmonary tract (8).

It is now well recognized that patients with CVID are also at risk for autoimmune, inflammatory, and nonmalignant lymphoproliferative complications as well as malignancies (9, 10). Patients with CVID and autoimmune, inflammatory, and nonmalignant lymphoproliferative complications driven by immune dysregulation are often labeled as having “complicated CVID” (CVIDc) (11). The reported frequencies of noninfectious clinical manifestations driven by immune dysregulation vary across published cohorts, which may in part be due to differences in referral patterns or geographic locations that affect the heterogeneity in the distribution of genetic drivers associated with CVID and other population characteristics across those cohorts (1). Monogenic causes and associations (e.g., *TACI* variants) have only been identified in approximately one-quarter to one-third of patients, ranging from as few as 3–4% in large registries that span several decades (12, 13) to 31–54% among three more recent, well-described, simultaneously analyzed cohorts (14). These varying frequencies for identification of genetic drivers in patients with CVID may be at least partially attributable to differences in patient ages, ethnicities, and degree of consanguinity across distinct cohorts, as well as variable availability of genetic testing across centers and lack of a standardized genetic panel for CVID diagnosis (1, 12, 13, 15).

Most patients with CVID are managed with Ig replacement therapy (IRT), which largely ameliorates the infectious risks, and antimicrobial therapies are prescribed as needed (2, 16). Malignancies are typically treated as per standard-of-care approaches for the specific cancer type (17). However, the treatment landscape lacks standardization regarding the noninfectious, nonmalignant lymphoproliferative, autoimmune, and inflammatory complications of immune dysregulation in patients with CVIDc. Different complications frequently coincide, suggesting common or at least overlapping etiologic drivers (12). Therefore, co-occurring complications of immune dysregulation may be amenable to a single-treatment approach, as they may share a common disease pathophysiology. Various immunosuppressive and immunomodulatory treatments are used, including corticosteroids, rituximab, sirolimus, abatacept, azathioprine, Janus-kinase (JAK) inhibitors, and others (17, 18), but none have been tested in randomized clinical trials of patients with CVIDc, and retrospective and observational data are quite limited. There is a need to integrate available information regarding the extensiveness of noninfectious and nonmalignant clinical manifestations driven by immune dysregulation in patients with CVIDc, including the current treatment landscape and factors which impact survival, to better understand the unmet clinical needs of patients. To address this need, a systematic literature review (SLR) was conducted to provide a comprehensive reference for the field summarizing the presenting features, genetic observations, frequencies of noninfectious and nonmalignant manifestations, immunosuppressive and immunomodulatory treatments used, and factors impacting survival. These data highlight the unmet need to develop treatments targeting noninfectious manifestations in patients with CVIDc to improve outcomes.

## Results

### Study selection

Literature searches were performed on April 15, 2024, with no limitations on date of publication. Study eligibility criteria were defined in terms of the population, interventions, comparisons, outcomes, and study design (PICOS) structure outlined in Table 1, which guided the identification and selection of studies relevant to the current SLR.

**Table 1.** Study eligibility criteria of the SLR

Criteria	Inclusion criteria	Exclusion criteria
Population	•Humans with CVID	•Studies with ≤10 patients (unless intervention was described)
		•Animal studies
		•In vitro studies
Interventions/comparators	•No restriction, as long as noninfectious, nonmalignant manifestations were reported	​
Outcomes	•Description of the clinical profile of patients, including:	•Studies focused on vaccine response or IRT
	o Median age and range of symptom onset (including for individual immune dysregulation manifestations when reported) and CVID diagnosis (separating pediatric and adult patients when reported)	
	o Genetic diagnoses	
	o Frequency of patients with:	
	Distinct immune dysregulation manifestations	
	Infections	
	Other reported manifestations	
	o Mortality and causes of death	
	•Description of the current treatment landscape for patients with CVID, including treatment agents and responses	
Study design	•Randomized controlled trials	•SLRs^a^
	•Nonrandomized clinical trials (e.g., single-arm trials)	•Narrative reviews
	•Prospective and retrospective observational studies	•Letters to editor, notes, and editorials
	•Case-control studies	•Case reports and case series (unless intervention was described)
Publication type	•Full-text publications	​
	•Conference abstracts/posters^b^	​
Language	•Studies published in English only	​
Time	•No time restriction	​

a Bibliographies of relevant systematic reviews were reviewed.

b Conference abstract/poster citations captured through search of main databases were excluded; they were searched separately in the Northern Light database or published proceeding from target conferences.

**Figure 1.**
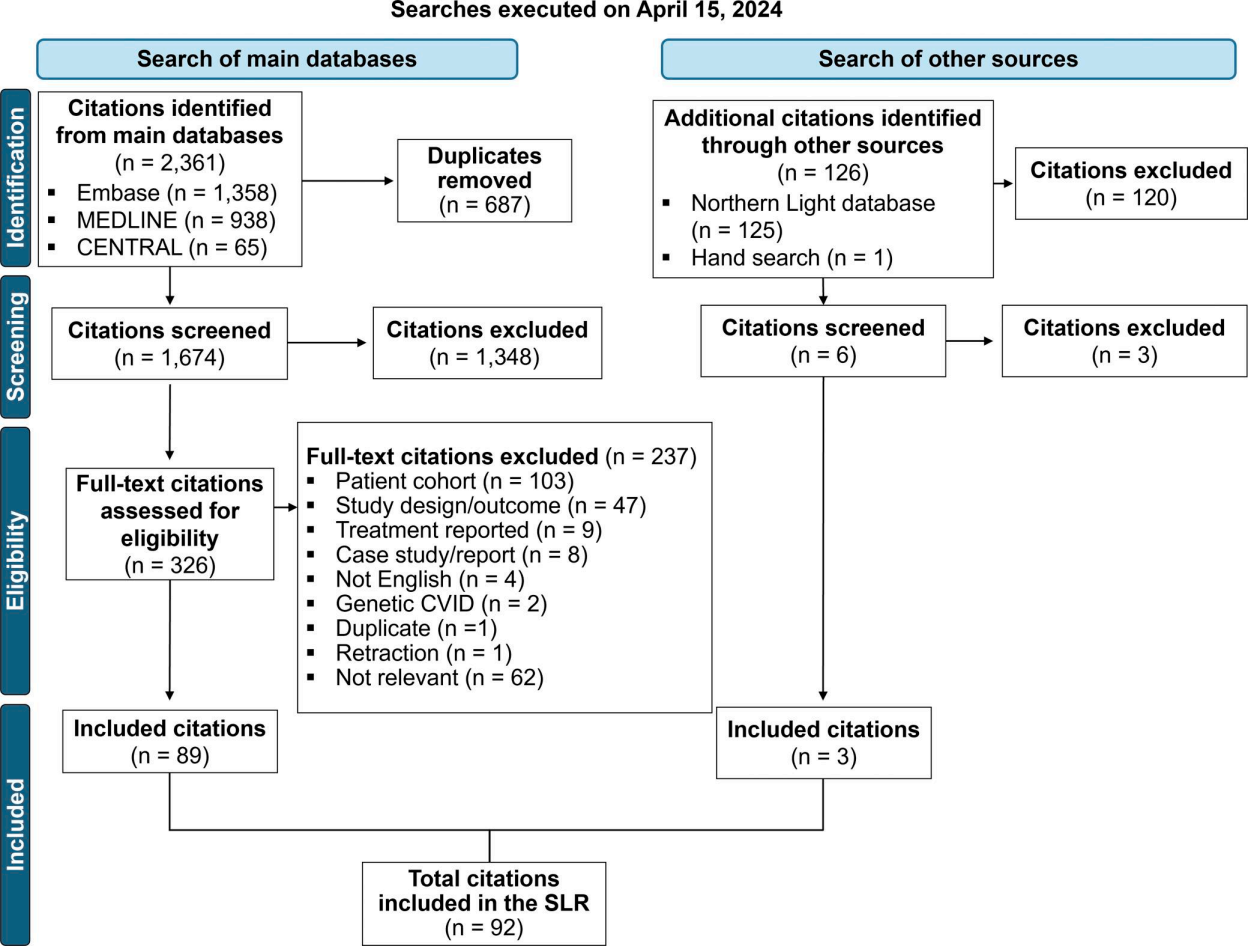
Study selection PRISMA flow diagram.

A total of 2,361 citations were identified through Excerpta Medica Database (Embase), Medical Literature Analysis and Retrieval System Online (MEDLINE), and Cochrane Central Register of Controlled Trials (CENTRAL). Of the total citations identified, 687 were duplicate citations and removed, and the remaining 1,674 citations were included for abstract and title screening. After screening the abstracts and titles, a further 1,348 citations were excluded, resulting in 326 citations included for full-text assessment. Of the 326 citations that underwent full-text screening, 237 were excluded for the following reasons (Table S1): cohort size or characteristics (103 citations), study design or reported outcomes (47 citations), case studies or reports (8 citations), IRT treatment only or no relevant treatment (e.g., omalizumab in asthmatic patients only) reported (9 citations), non-English publication (4 citations), reporting genetic CVID only (2 citations), duplicate publication (1 citation), retraction (1 citation), and missing relevant information (62 citations). An additional 3 citations were identified through other sources (2 conference abstracts via Northern Light database search and 1 full-text publication via hand search). In total, 92 citations were included in the data extraction phase as shown in the study selection Preferred Reporting Items for Systematic Reviews and Meta-Analyses (PRISMA) flow diagram (Fig. 1).

### Literature characteristics

Out of the 92 citations included in the SLR, 9 studies reported information from cohorts composed of more than 500 patients (10, 12, 13, 15, 19, 20, 21, 22, 23), 36 studies had between 101 and 500 patients (1, 8, 9, 24, 25, 26, 27, 28, 29, 30, 31, 32, 33, 34, 35, 36, 37, 38, 39, 40, 41, 42, 43, 44, 45, 46, 47, 48, 49, 50, 51, 52, 53, 54, 55, 56), 24 studies had between 51 and 100 patients (14, 57, 58, 59, 60, 61, 62, 63, 64, 65, 66, 67, 68, 69, 70, 71, 72, 73, 74, 75, 76, 77, 78, 79), 18 studies had between 11 and 50 patients (80, 81, 82, 83, 84, 85, 86, 87, 88, 89, 90, 91, 92, 93, 94, 95, 96, 97), and 5 studies had <10 patients but reported on treatments related to noninfectious and nonmalignant manifestations (i.e., not only IRT) (Table S2) (98, 99, 100, 101, 102). Twenty of the 92 studies reported genetic findings, 89 reported clinical manifestations, 16 reported treatments administered, and 33 reported survival outcomes. The geographic locations of patients reported in the 92 studies were predominantly Europe (52%) and North America (22%), followed by Middle East and North Africa (17%), Oceana (3%), South America (2%), Asia (1%), and a combination of geographic locations (2%).

### Disease presentation and diagnostic delay

Patient ages at time of onset, CVID diagnosis, and study; diagnostic delay; and duration of follow-up were reported as either a median or mean value across the studies (Table S2). The median and mean ages at time of disease onset were 18.0 years (range, 2.0–43.4 years; 37 studies) and 18.1 years (range, 2.3–29.8 years; 8 studies), respectively. The median and mean ages at time of diagnosis were 28.0 years (range, 5.0–52.0 years; 50 studies) and 30.4 years (range, 5.5–58.6 years; 13 studies), respectively. The median and mean diagnostic delays were 5.0 years (range, 1.0–46.5 years; 26 studies) and 9.5 years (range, 4.4–14.0 years; 8 studies), respectively. At the time of study, the median and mean patient ages were 44.0 years (range, 9.0–65.6 years; 39 studies) and 41.6 years (range, 12.8–54.1 years; 16 studies), respectively. The median and mean follow-up durations were 9.7 years (range, 2.1–19.5 years; 17 studies) and 10.0 years (range, 5.2–24.5 years; 7 studies), respectively.

Of the publications reporting clinical manifestations of immune dysregulation, 10 provided percentages of patients with infections and with immune dysregulation. Infections only were reported in a median of 25.0% of patients (range, 18.0–54.0%; 5 studies), and infections in general (with or without other complications) were reported in a median of 52.9% of patients (range, 33.6–100.0%; 6 studies). Immune dysregulation (with or without infections) was reported in a median of 62.9% of patients (range, 23.0–82.1%, 10 studies). Of these 10 studies, 2 reported the presenting manifestations. Infections were reported as the presenting manifestation in 63.2–89.0% of patients, while immune dysregulation manifestations were reported as the presenting feature in 33.2–42.5% of patients (28, 61).

### Genetic variants associated with CVID phenotype

**Figure 2.**
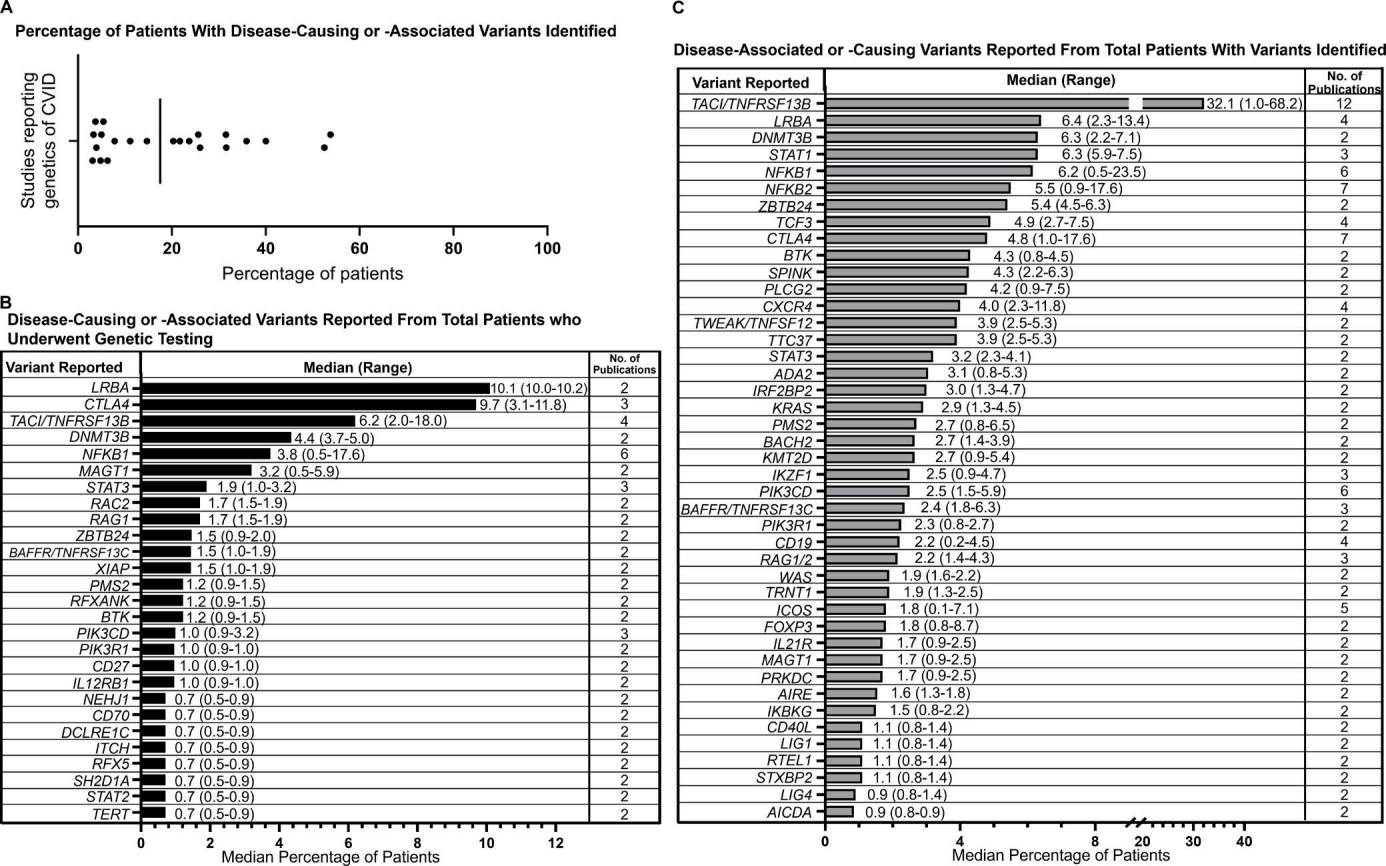
**Gene variants reported in two or more publications. (A)** Percentage of patients with disease-causing or -associated variants identified from all patients with CVID. Each dot represents an individual study (*n*, 19 studies); the line represents the calculated median. **(B)** Median percentages of patients with disease-causing or -associated gene variants determined from total patients who were genetically tested. **(C)** Median percentages of patients with disease-causing or -associated variants determined from total number of patients with gene variants identified.

Twenty publications reported genetic findings, 1 of which reported that all patients had only benign variants or variants of uncertain significance (VUSs) (28) and was therefore excluded from collation. In total, 42 patients across 3 studies were excluded due to having variants characterized as benign or uncertain significance. Frequencies of the remaining genetic variants identified were either reported directly from the respective publications or manually calculated from the information presented. The median percentage of patients who had a disease-causing or -associated genetic variant identified was 17.5% (range, 3.1–53.8%; 19 studies) (Fig. 2 A).

Seven publications reported the percentages of genetic observations based on the total number of patients who underwent genetic testing, and 12 publications reported percentages based on the total number of patients who had a genetic variant identified. Given that the denominator was distinct for each group, we summarized and stratified genetic findings separately (Table S2 and Fig. 2).

Out of 7 publications reporting gene variants related to the total number of patients tested, disease-causing or -associated variants in 27 genes were reported by 2 or more articles. Genetic variants in *NFKB1* were the most often reported, with 6 studies reporting observations of *NFKB1* variants among patients with CVID, followed by *TACI* (4 studies), *CTLA4* (3 studies), and *ICOS* (3 studies). The highest median frequencies of observed or associated genetic drivers were 10.1% for *LRBA* (range, 10.0–10.2%; 2 studies), 9.7% for *CTLA4* (range, 3.1–11.8%; 3 studies), 6.2% for *TACI* (range, 2.0–18.0%; 4 studies), 4.4% for *DNMT3B* (range, 3.7–5.0%; 2 studies), and 3.8% for *NFKB1* (range, 0.5–17.6%; 6 studies) (Fig. 2 B). Out of the 12 publications reporting gene variants in relation to the total number of patients with variants identified, disease-causing or -associated variants in 43 genes were reported by 2 or more articles. *TACI* (12 studies), *NFKB2* (7 studies), *CTLA4* (7 studies), *NFKB1* (6 studies), and *PIK3CD* (6 studies) were the most commonly reported. The highest median frequencies of observed or associated genetic drivers were 32.1% for *TACI* (range, 1.0–68.2%; 12 studies), 6.4% for *LRBA* (range, 2.3–13.4%; 4 studies), 6.3% for *DNMT3B* (range, 2.2–7.1%; 2 studies), 6.3% for *STAT1* (range, 5.9–7.5%; 3 studies), 6.2% for *NFKB1* (range, 0.5–23.5%; 6 studies), and 5.5% for *NFKB2* (range, 0.9–17.6%; 7 studies) (Fig. 2 C).

### Noninfectious, nonmalignant clinical manifestations of immune dysregulation

Clinical manifestations of immune dysregulation were pervasive in the evidence base. 89 studies reported clinical manifestations, and 86 of these reported noninfectious, nonmalignant clinical manifestations of immune dysregulation. We summarized information regarding lymphoproliferative, autoimmune, and inflammatory manifestations by organ systems and subspecialty areas for which data were available from at least 10 publications and for which at least 3 publications reported a specific manifestation, unless the studies were focused on a subset of patients (e.g., a report of patients with nodular regenerative hyperplasia [NRH] of the liver).

### Lymphoproliferative manifestations

**Figure 3.**
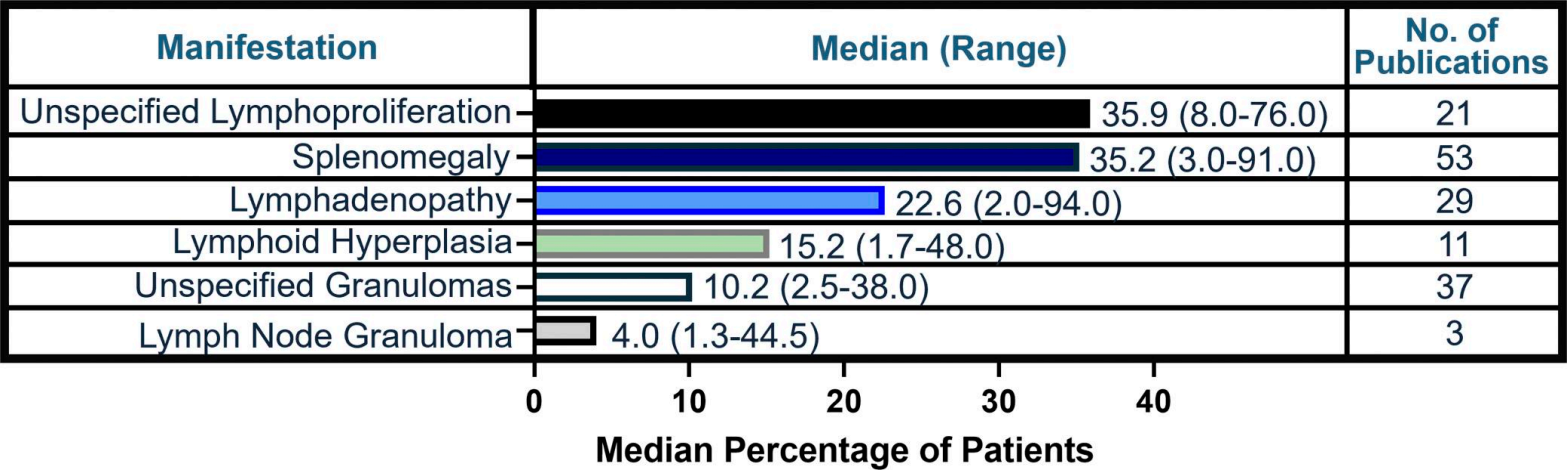
**Frequency of lymphoproliferative manifestations in literature.** Calculated median (range) percentage of patients and number of publications reported for each lymphoproliferative manifestation.

Unspecified lymphoproliferation was the most frequent manifestation, reported in a median of 35.9% of patients (range, 8.0–76.0%; 21 studies) (Fig. 3 and Table S3). Splenomegaly was the most reported lymphoproliferative manifestation among studies included in the evidence base (median, 35.2%; range, 3.0–91.0%; 53 studies). Lymphadenopathy was reported in a median of 22.6% of patients (range, 2.0–94.0%; 29 studies). 37 studies reported unspecified granulomas in a median of 10.2% of patients (range, 2.5–38.0%).

### Digestive system manifestations

**Figure 4.**
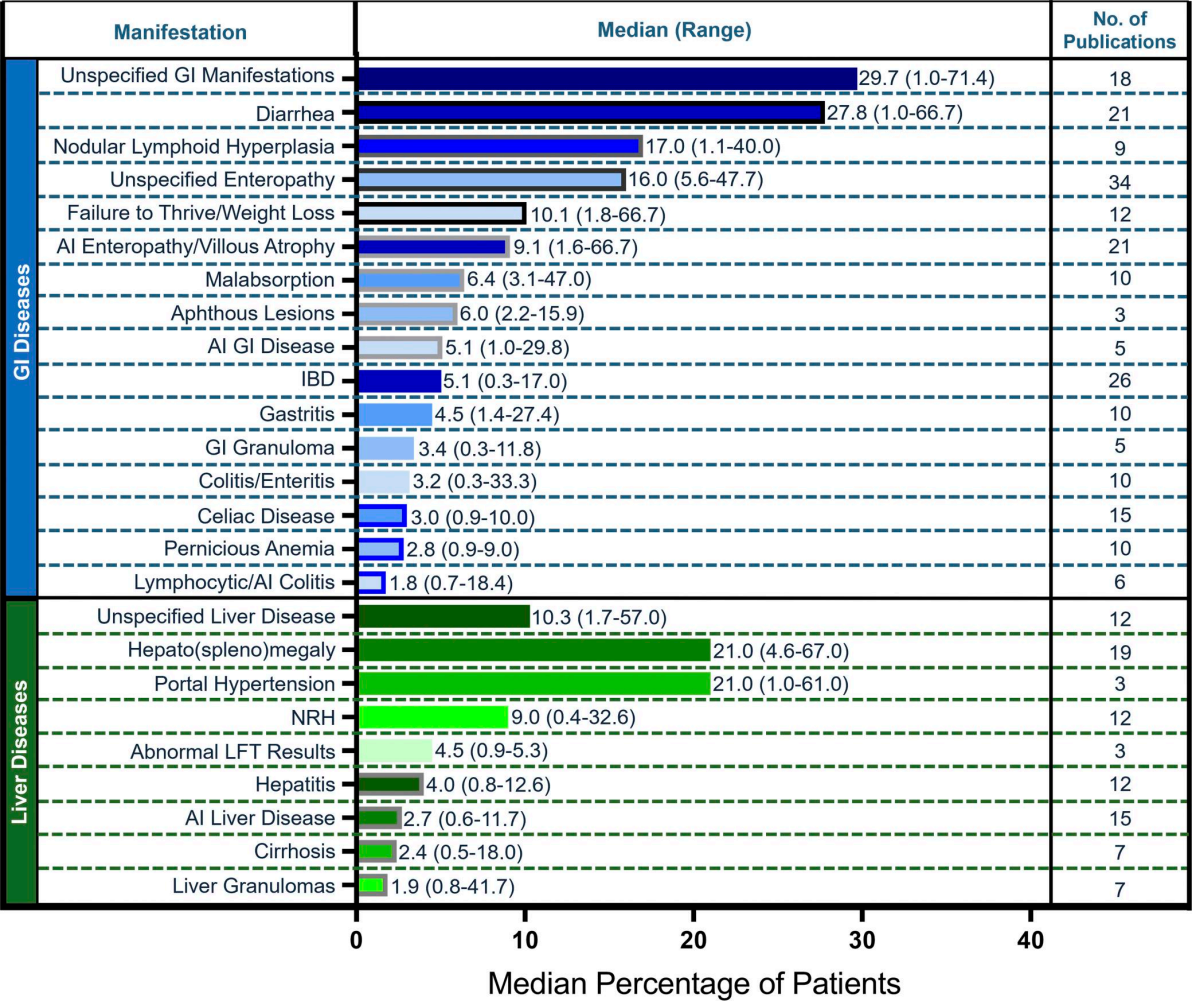
**Frequency of digestive system manifestations in literature.** Calculated median (range) percentage of patients and number of publications reported for each GI manifestation (blue bars) and liver manifestation (green bars). AI, autoimmune; LFT, liver function test.

Gastrointestinal (GI) manifestations were reported in 56 of the publications included in the evidence base (Fig. 4 and Table S3). Studies most frequently reported unspecified enteropathy (34 studies), inflammatory bowel disease (IBD; 26 studies), diarrhea (21 studies), and autoimmune enteropathy or villous atrophy (21 studies). The most frequently reported GI manifestations among patients with CVID included diarrhea (median, 27.8%; range, 1.0–66.7%), nodular lymphoid hyperplasia (median, 17.0%; range, 1.1–40.0%), unspecified enteropathy (median, 16.0%; range, 5.6–47.7%), failure to thrive or weight loss (median, 10.1%; range, 1.8–66.7%), and autoimmune enteropathy or villous atrophy (median, 9.1%; range, 1.6–66.7%). Fewer than 10% of patients had malabsorption, aphthous lesions, autoimmune GI disease, IBD, gastritis, GI granuloma, colitis or enteritis, celiac disease, pernicious anemia, and lymphocytic or autoimmune colitis.

Liver manifestations were reported in 50 studies, and hepato(spleno)megaly was the most reported manifestation among both studies and patients (median, 21.0%; range, 4.6–67.0%; 19 studies) (Fig. 4 and Table S3). Other frequently reported manifestations among patients included portal hypertension (median, 21.0%; range, 1.0–61.0%; 3 studies) and NRH (median, 9.0%; range, 0.4–32.6%; 12 studies). Abnormal liver function test results, hepatitis, liver granulomas, autoimmune liver disease, cirrhosis, and other liver manifestations were reported in <5% of patients.

### Lung manifestations

**Figure 5.**
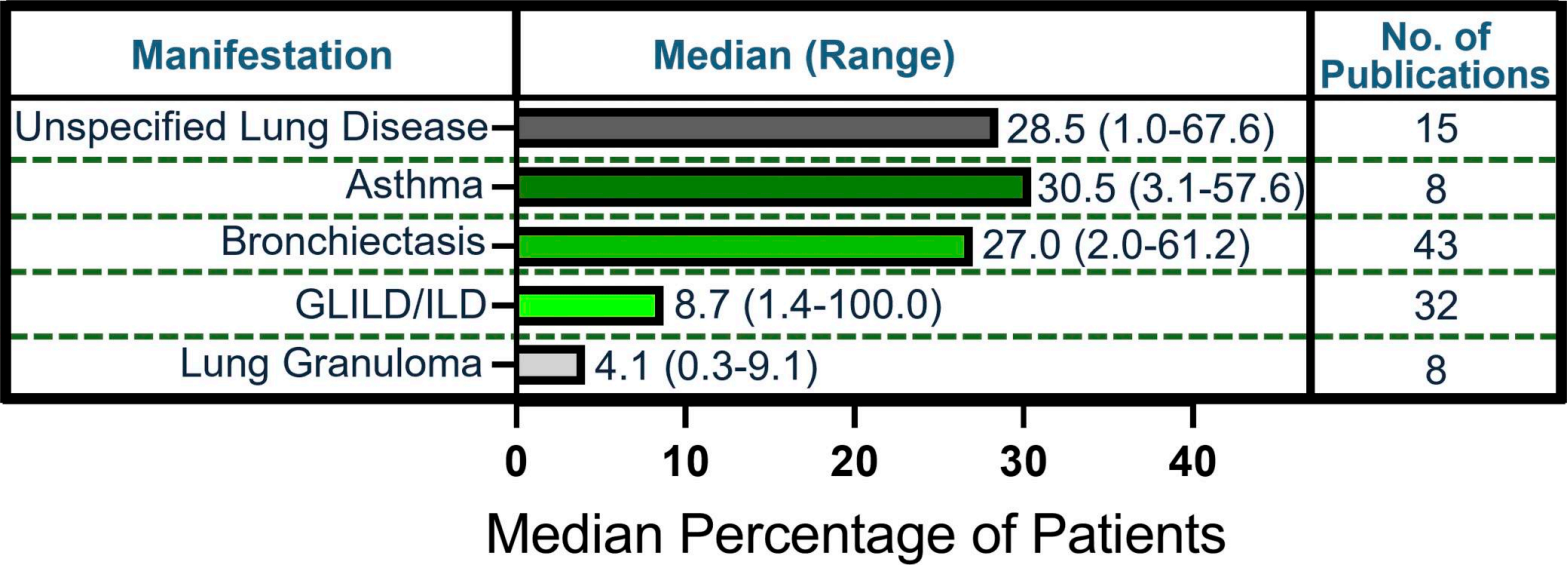
**Frequency of lung manifestations in literature.** Calculated median (range) percentage of patients and number of publications reported for each lung manifestation.

59 studies reported patients with lung manifestations (Fig. 5 and Table S3). The most common among patients were asthma (median, 30.5%; range, 3.1–57.6%; 8 publications) and bronchiectasis (median, 27.0%; range, 2.0–61.2%; 43 publications). Granulomatous lymphocytic interstitial lung disease (GLILD) or interstitial lung disease (ILD) were reported in 32 studies in a median of 8.7% of patients (range, 1.4–100.0%). Eight studies reported lung granulomas in 4.1% of patients (range, 0.3–9.1%).

### Autoimmune cytopenias

**Figure 6.**
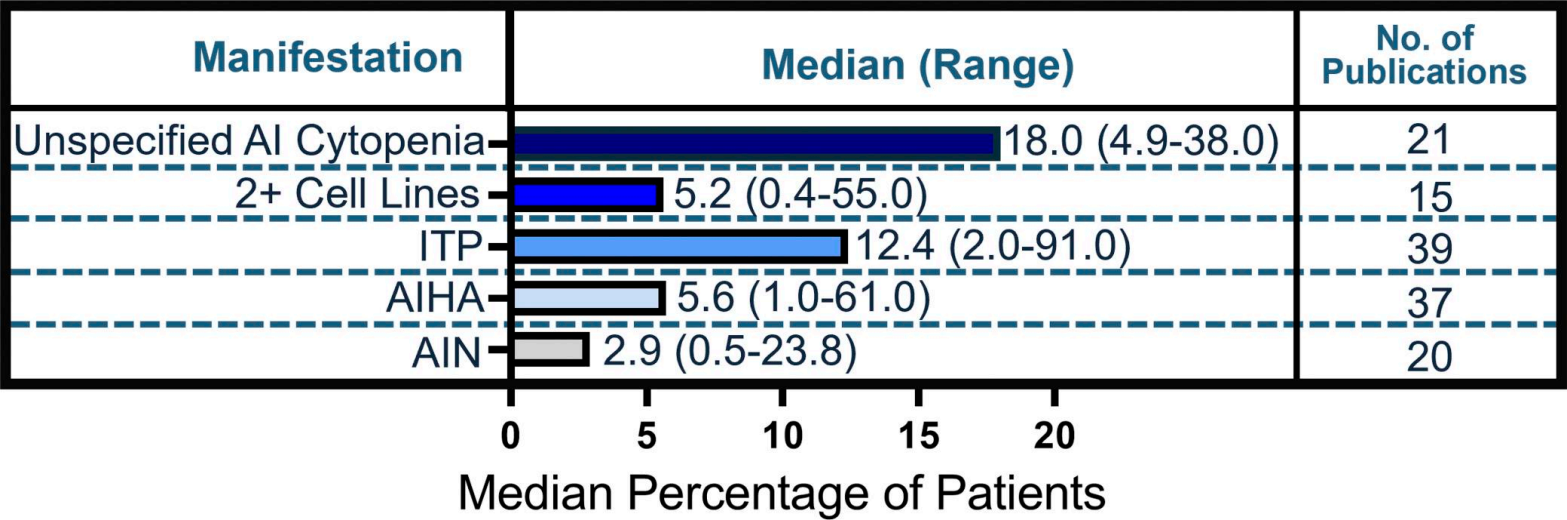
**Frequency of autoimmune cytopenias in literature.** Calculated median (range) percentage of patients and number of publications reported for each cytopenia. AI, autoimmune; AIHA, autoimmune hemolytic anemia; AIN, autoimmune neutropenia; ITP, idiopathic thrombocytopenia.

54 studies reported autoimmune cytopenias (Fig. 6 and Table S3), 7 of which reported both unspecified and specified autoimmune cytopenias. Unspecified autoimmune cytopenias were most frequently reported among patients (median, 18.0%; range, 4.9–38.0%; 21 studies). Of specific cytopenias, immune thrombocytopenia was most frequently reported among patients and studies (median, 12.4%; range, 2.0–91.0%; 39 studies). Cytopenias affecting ≥2 cell lines or Evans syndrome were reported in a median of 5.2% of patients (range, 0.4–55.0%; 15 studies).

### Additional noninfectious, nonmalignant manifestations

**Figure 7.**
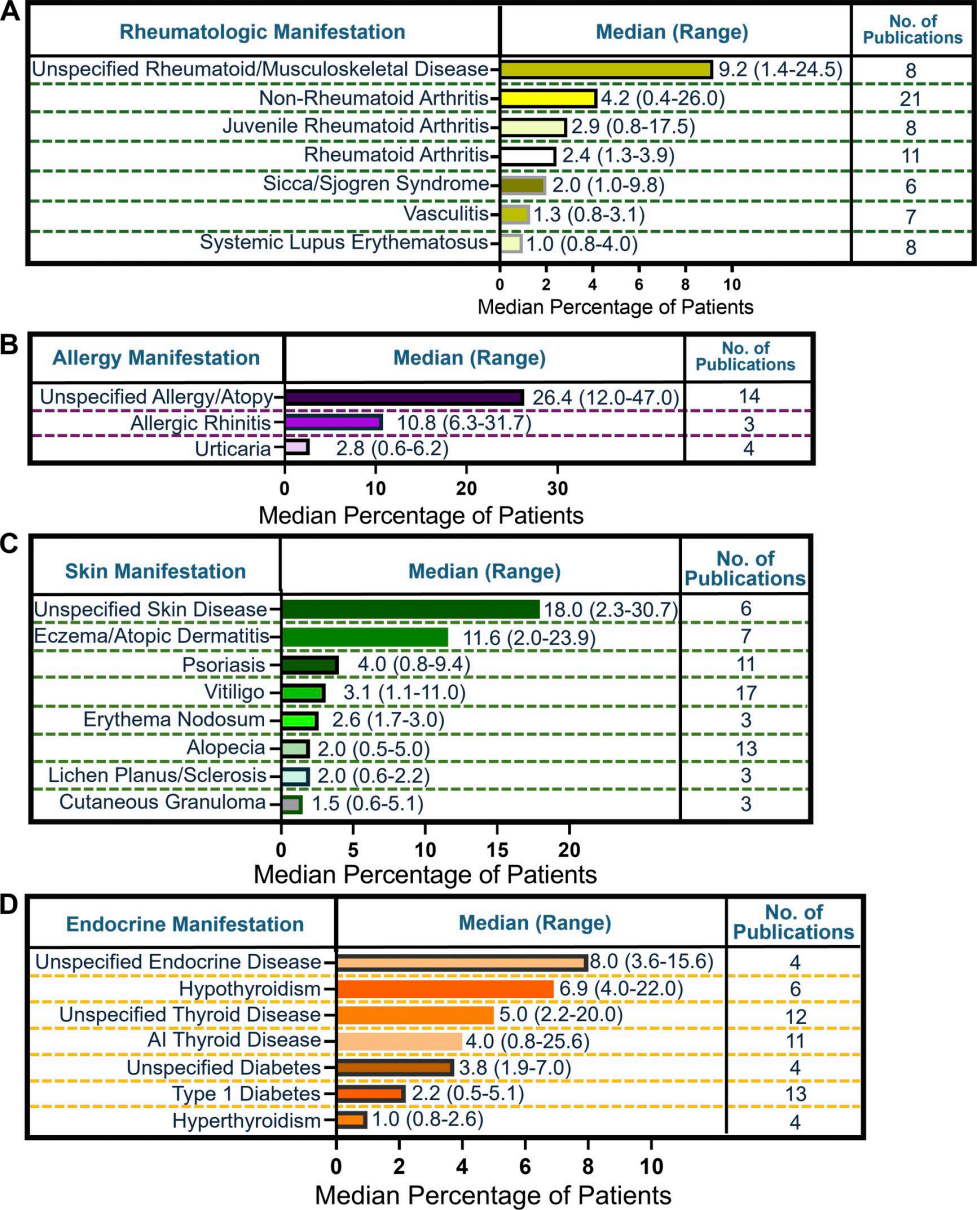
**Frequency of additional noninfectious nonmalignant manifestations reported in literature. (A)** Calculated median (range) percentage of patients and number of publications reported for each rheumatologic manifestation. **(B)** Calculated median (range) percentage of patients and number of publications reported for each allergy manifestation. **(C)** Calculated median (range) percentage of patients and number of publications reported for each skin manifestation. **(D)** Calculated median (range) percentage of patients and number of publications reported for each endocrine manifestation. AI, autoimmune.

Rheumatologic manifestations were reported across 39 studies and included non-rheumatoid arthritis (non-RA), juvenile RA, RA, Sicca or Sjogren’s syndrome, and others (Fig. 7 A and Table S3). Across all studies describing specified rheumatologic manifestations, non-RA was the most frequently reported manifestation among studies and patients (median, 4.2%; range, 0.4–26.0%; 21 studies).

Allergic manifestations were reported across 20 studies, including unspecified allergy reported in a median of 26.4% of patients (range, 12.0–47.0%; 14 studies) (Fig. 7 B and Table S3). Allergic rhinitis was the most commonly reported specified manifestation among patients (median, 10.8%; range, 6.3–31.7%; 3 studies). Urticaria was reported in a median of 2.8% of patients (range, 0.6–6.2%; 4 studies).

Skin manifestations were reported in 33 studies (Fig. 7 C and Table S3), and unspecified skin disease was the most common manifestation (median, 18.0%; range, 2.3–30.7%; 6 studies). Eczema or atopic dermatitis was reported in a median of 11.6% of patients (range, 2.0–23.9%; 7 studies). Psoriasis and vitiligo were reported in 4.0% (range, 0.8–9.4%; 11 studies) and 3.1% (range, 1.1–11.0%; 17 studies) of patients, respectively.

Endocrine complications were reported across 38 studies, including unspecified endocrine complications in a median of 8.0% of patients (range, 3.6–15.6%; 4 studies) (Fig. 7 D and Table S3). Type 1 diabetes was reported in 13 studies, making it the most commonly reported manifestation (median, 2.2%; range, 0.5–5.1%). Thyroid diseases were also commonly reported in patients across 28 studies and included hypothyroidism (median; 6.9%; range, 4.0–22.0%; 6 studies), autoimmune thyroid disease (median, 4.0%; range, 0.8–25.6%; 11 studies), and hyperthyroidism (median, 1.0%; range, 0.8–2.6%; 4 studies).

### Treatment information

10 studies reported treatments prescribed across the CVID patient cohorts. Treatments, with the exclusion of IRT and antibiotics, reported in >2 publications were included in Table 2. The treatments were summarized as a calculated median of the percentages of patients who were administered each specific drug at any time throughout their disease course across all the studies (Table S3). For each study, percentages were either directly provided or they were calculated manually based on the number of patients who were administered a specific therapy out of the total number of patients in the respective cohort. Steroids, including prednisone, were reported in a median of 56.4% (range, 32.9–85.7%) of patients across nine studies. Rituximab was reported in a median of 12.2% (range, 5.5–34.8%; 7 studies). Several other immunosuppressive and immunomodulatory therapies were reported in up to eight studies. Mycophenolate and azathioprine were reported in medians of 7.4% (range, 1.8–13.2%; 6 studies) and 7.0% (range, 3.6–28.6%; 8 studies) of patients, respectively, while other agents were reported in a median of <5% of patients. Thalidomide, sulfasalazine, adalimumab, obinutuzumab, ustekinumab, vedolizumab, sertraline, and danazol were all additionally reported in one study each and used in 1 to 2 patients per study (not shown in table).

**Table 2.** Treatments reported in three or more studies (excluding IRT and antibiotics)^a^

Treatment type	Median percentage of patients (range)	No. of publications
Steroids	56.4 (32.9–85.7)	9
Rituximab	12.2 (5.5–34.8)	7
Mycophenolate	7.4 (1.8–13.2)	6
Azathioprine	7.0 (3.6–28.6)	8
Antimalarials	4.4 (2.6–7.3)	4
TNF-α inhibitors	3.7 (1.5–9.0)	5
Methotrexate	3.6 (0.8–15.4)	5
Tacrolimus	3.0 (2.6–3.0)	3
Cyclophosphamide	2.6 (0.4–10.9)	3
Abatacept	2.4 (0.4–3.0)	4
Sirolimus	2.0 (0.8–7.5)	4
Cyclosporine	1.7 (0.8–2.2)	4

TNF-α, tumor necrosis factor alpha.

a Six case studies reporting monotherapies were excluded, as all patients were administered the same treatment.

### Survival outcomes

We examined the reported survival and causes of death among patients with CVID, including the impact of noninfectious and nonmalignant complications. 31 studies reported the percentage of deceased patients in the CVID cohorts (Table S3). The calculated median percentage of deceased patients was 15.6% (range, 3.0–56.0%; 31 studies). The median age of death among deceased patients with CVID was 44.0 years (range, 24.0–72.0 years; 7 studies).

**Figure 8.**
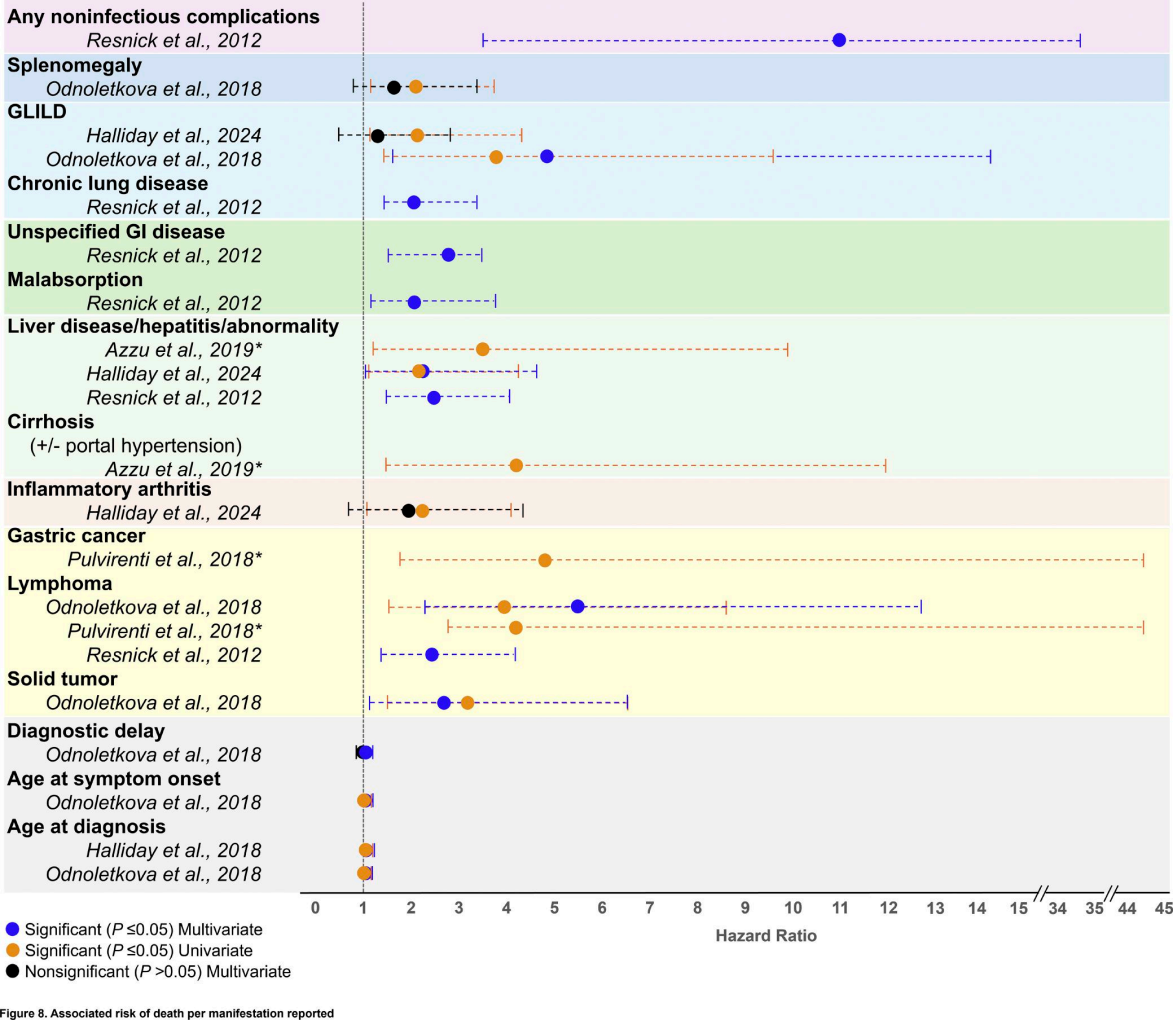
**Associated risk of death per manifestation reported.** Manifestations with HRs reported to be significant (P ≤ 0.05) for at least one analysis (univariate or multivariate). All analyses (significant and nonsignificant) were included in the figure for those manifestations with at least one significant HR. A list of all manifestations reported with an HR, including those not observed to have statistical significance, is provided in Table S4. Dashed lines represent 95% CIs. *Assumption is that analysis was univariate. References cited: (23) Odnoletkova et al., 2018; (36) Halliday et al., 2024; (41) Pulvirenti et al., 2018; (44) Resnick et al., 2012; (58) Azzu et al., 2019.

The impact of several noninfectious manifestations on survival was evaluated using reported hazard ratios (HRs) per manifestation across five studies, via both univariate and multivariate analyses (Fig. 8 and Table S4). The presence of any noninfectious manifestation in patients with CVID was associated with the highest significant mortality risk (HR, 11.0) as reported in a large single study cohort with patients followed for up to four decades (44). Of the specific noninfectious manifestations reported with significant HRs, multivariate analyses from one study reported that lymphoma (HR, 5.5; 95% confidence interval [CI], 2.4–12.7) and GLILD (HR, 4.9; 95% CI, 1.6–14.4) were associated with the highest mortality risks (23). Liver disease also conveyed a significant negative impact on survival (Fig. 8). Autoimmune cytopenias, bronchiectasis, and enteropathy were not reported to significantly affect survival (Table S4). Increasing age at symptom onset and increasing age at CVID diagnosis were both associated with significant increased risk of mortality, as was diagnostic delay when adjusted for the age of symptom onset (HR, 1.04; 95% CI, 1.02–1.06; P = 0.0003) but not when adjusted for the age at CVID diagnosis (23).

17 studies reported the causes of death among patients with CVID (Table S3). The most frequent causes of death were respiratory or lung diseases (median, 20.0%; range, 5.6–37.5%; 11 studies), infectious diseases (median, 18.8%; range, 2.0–40.0%; 14 studies), and lymphoma (median, 13.3%; range, 2.0–21.7%; 9 studies). Infectious diseases included pneumonia and cytomegalovirus, among other causes. Of the 11 studies reporting nonlymphoid or unspecified malignancy as a cause of death, 7 reported unspecified malignancies, and 4 reported specific malignancies, including breast, GI tract, liver, lung, pancreatic, uterine, and ovarian cancers. Cytopenias, including anemia, neutropenia, and bone marrow aplasia, were reported as the cause of death across four studies in a median of 4.3% of patients (range, 3.5–9.0%). Other less commonly reported causes of death included Kawasaki disease, fever with unknown origin, and allogenic hematopoietic cell transplant complications, each reported in one or two patients across three different studies.

## Discussion

To the best of our knowledge, this study represents the first extensive SLR conducted to collate reports across the CVID literature and define the profile of patients with CVIDc who display noninfectious and nonmalignant clinical manifestations of immune dysregulation. Our work reveals the pervasiveness of lymphoproliferative, autoimmune, and inflammatory complications and the negative impact they convey on the outcomes of patients with CVIDc. Importantly, several points of intervention were identified that could lead to improved patient outcomes, including expediting the time to diagnosis, identification of genetic causes, and better treatment interventions targeting immune dysregulation.

First, the observed median diagnostic delay of 5.0 years presents an obvious call to action regarding expediting patient diagnosis (8, 9, 14, 22, 23, 24, 32, 34, 39, 46, 47, 49, 50, 51, 52, 53, 57, 66, 67, 76, 81, 82, 88, 89). Odnoletkova et al. demonstrated that each year of diagnostic delay was associated with a striking 4% increase of the risk of death in an analysis of 2,700 patients with CVID included in the European Society for Immunodeficiencies registry (23). Remarkably, the authors also observed that diagnostic delay had not decreased over a timespan of more than 30 years, suggesting that educational awareness efforts and diagnostic improvements have not yet made a measurable impact on timely diagnoses. These data strongly support the need for a focus on novel diagnostic approaches. The emerging implementation of validated automated decision support tools is one approach that holds promise to decrease the diagnostic delay observed in more than 80% of patients with CVID (23, 103). Given that approximately one-third of patients with CVID will present with manifestations of immune dysregulation only (13) in the absence of infectious complications, it is therefore key that algorithms, along with healthcare providers, are able to detect patients with CVIDc in the absence of a presenting infectious phenotype.

Second, the observation of underlying disease-causing or disease-associated genetic variants in a median of 18% of patients with CVID makes a case for performing genetic testing in all patients suspected to have CVID. Knowledge of an underlying genetic disorder allows for prompt screening assessment and subsequent intervention as needed for particular genetic disorder–associated clinical manifestations of immune dysregulation, such as central nervous system disease in CTLA-4 haploinsufficiency (104). Although monogenic causes of CVID were not the main focus of this SLR (i.e., studies reporting only on a specific monogenic cause were excluded), the most reported CVID-causing or -associated genetic variants observed across 19 publications included *TACI*, *NFKB1*, *CTLA4*, *PIK3CD*, *NFKB2*, and *LRBA*; the genes with the highest median frequencies among patients with identified genetic variants included *TACI*, *LRBA*, *DNMT3B*, *STAT1*, *NFKB1*, and *NFKB2*. Unsurprisingly, many of these are strongly characterized by immune dysregulation (105, 106, 107, 108, 109).

Among the 19 studies from which the genetic data were extracted and collated, 8 were from European centers, 6 from North American centers, 3 from Middle Eastern or North African centers, and 1 was from Oceana. Of note, Abolhassani et al. reported on three cohorts: North American (United States), European (Sweden), and Middle Eastern or North African (Iran). The three most common genetic variants observed in the different cohorts published by Abolhassani et al. were *TACI*, *NFKB1*, and *CTLA4* in the North American cohort; *LRBA*, *TACI*, and *FOXP3* in the European cohort; and *LRBA*, *DNMT3B*, and *BTK* in the Middle Eastern or North African cohort (15). It is generally accepted that *NFKB1* is the most frequent cause of monogenic CVID in Europe and North America (1, 15, 110), whereas the median frequency among studies included in this global SLR reported *NFKB1* as the fifth most frequent CVID-causing or -associated gene (with a high upper range of frequency). Overall, this information is not surprising given the normal variation observed in genetic variants across different geographic populations based on their demographics and the extent of consanguinity. A recent case-controlled CVID study published after the date of our literature search reported a similar distribution of genetic disorders among patients and included variants in *NFKB1*, *TACI*, *CTLA4*, *LRBA*, *STAT3*, and *ICOS* (111). Additionally, among patients with predominantly antibody deficiency, which includes CVID, variants in *TNFRSF13B*, *NFKB1*, and *CTLA4* were also commonly observed and accounted for 72.2% of patients with a genetic driver or association identified in a German cohort (112).

To date, there are over 20 genes classified by the International Union of Immunological Societies expert committee as CVID disease-causing or -associated (7). The current SLR identifies over 50 genes that have been reported across the included CVID cohort studies to be disease-causing or -associated. Given that CVID is heterogenous, we do not suggest that all of these genetic variants indeed “cause CVID,” but rather that CVID is a clinical diagnosis, and phenotypic designation is associated with a number of genetic changes. Patients with a variety of underlying genetic disorders may develop immune dysregulation that results in clinical manifestations that are consistent with a CVIDc phenotype (13). Whether monogenic CVID disorders should be referred to as CVID or conceptualized individually based on their genetic driver will continue to evolve in the field (4).

Third, the recognition of the pervasive nature of noninfectious, nonmalignant clinical manifestations of immune dysregulation emphasizes the unmet need to widen efforts to develop novel therapeutics directed against these complications. Noninfectious manifestations were reported across 89 studies of patients with CVID, and yet no regulatory agency–approved therapeutics exist to address the treatment needs of patients with CVIDc with clinical manifestations driven by their immune dysregulation. Not surprisingly, steroids were the most common treatment reported among all the CVIDc cohorts and were prescribed in over half of the CVIDc population included in the current SLR. A variety of other immunosuppressive agents were also individually used in 2–12% of patients. Remarkably, studies reporting newer therapies such as JAK inhibitors were mostly lacking in the current SLR. This may be due to the exclusion of studies reporting only on a single monogenic cause of CVID (e.g., patients with identified pathogenic *JAK/STAT* variants) or simply reflect the more recent approval timeframes for JAK inhibitors and lack of sufficient time for current use to be accurately reflected in the literature.

Most importantly, the data make it clear that the noninfectious, nonmalignant clinical manifestations of immune dysregulation impart a large clinical burden, negatively impacting patients with CVIDc. Taken together, patients with CVIDc with any clinical manifestation of immune dysregulation have an 11-times greater risk of death than patients with CVID presenting with infectious manifestations alone, based on a large cohort of patients with CVID who were followed for up to four decades (44). The significant negative impacts on survival of specific individual manifestations are most readily apparent with GLILD and liver disease. Additional manifestations were also observed to impact survival as demonstrated by more limited univariate analyses. The increased mortality risk observed with the presence of “any” clinical manifestation of immune dysregulation may be due to the co-occurrence of immune dysregulation–driven CVIDc manifestations in individual patients. For example, in an unbiased network clustering analysis, “lymphoproliferative” groupings were identified that included splenomegaly, NRH, autoimmune hepatitis, GLILD, granulomas, and cytopenias, suggesting a shared endotype among a subset of patients with CVIDc that drives their higher mortality risk (12). In addition to the mortality burden of disease, it must be remembered that the myriad of lymphoproliferative, autoimmune, and inflammatory manifestations associated with CVIDc also impart an obvious high burden of morbidity to patients with CVIDc, adding further support to the large unmet medical need in this population.

Our data collated from the literature align with the recent study of 497 patients with CVID who were treated in Germany that was published after the current SLR cutoff date (111). In the German cohort, patients with CVIDc had higher mortality compared to those with an infectious phenotype alone, with an odds ratio of 3.1 (P = 0.01). A case-control analysis revealed that hepatopathy and severe enteropathy were significantly associated with increased mortality. Uniquely, the authors demonstrated that CD4^+^ T cell counts <400 cells per μl was associated with death among patients with CVIDc. It is unknown whether CD4^+^ T cell lymphopenia results from diminished production, consumption related to splenomegaly and/or hepatopathy, systemic immunosuppressive treatments, or a combination of these or other factors that may confound the association.

Another important aspect that warrants consideration regarding mortality in patients with CVIDc is that immune dysregulation often necessitates chronic treatment with immunosuppressive agents, thereby potentially exacerbating the immunocompromised state of patients. This additional burden may contribute to the increased morbidity and mortality observed in this subgroup and could partly explain why immune dysregulation is identified as a risk factor for mortality. These findings underscore the urgent need to reduce diagnostic delays, increase awareness of immune dysregulation manifestations, enhance access to genetic diagnosis globally, and develop targeted therapies that can mitigate the risks associated with broad immunosuppression. Given the genetic heterogeneity among patients with CVIDc, it remains to be seen if specific genetic defects convey risks of lymphoproliferative, autoimmune, and inflammatory complications via unique impacts on immunologic/cellular pathways, which would optimally benefit from uniquely tailored precision therapies, or if clusters of patients with diverse genetically defined and undefined drivers may share a common convergent pathophysiologic mechanism of disease that would align to a shared targeted treatment approach. The latter scenario could be particularly relevant to patients within the lymphoproliferative endotype group. Notably, it was recently demonstrated that patients with diverse monogenic forms of CVID, including activated PI3K delta syndrome, loss-of-function *NFKB1* variants, and pathogenic *CTLA4* variants, share dysregulated CD4^+^ T cell expansion and transitional B cell expansion, including increases in autoreactive 9G4^+^ B cells (113, *Preprint*). These observations suggest a common break in B cell tolerance, shared across diverse monogenic drivers of CVIDc, that may ultimately inform common pathways for immune modulation. As most patients with CVIDc remain without a known genetic etiology, common pathways for immune modulation may ultimately yield the largest clinical benefit across all CVIDc.

The SLR had several limitations. As the evidence base is continually expanding, any relevant studies published after the search date of April 15, 2024, were not captured in this SLR. In addition, any studies not yet indexed in the databases at the time of the search may not have been included in the publications extracted. The evidence presented here is limited to the way information was reported across the publications (e.g., different terminology for the same clinical parameter), leading to potential inconsistencies. Also, due to the inclusion requirement of reporting on CVID cohorts with more than 10 patients, relevant studies with smaller cohort sizes were not included. Patients may have been reported in multiple cohorts; however, duplicate entries could not be accounted for. As such, patients may be counted in multiple studies contributing to increased bias in the results. Bias towards cohorts with higher frequencies of immune dysregulation manifestations may have also been introduced with the exclusion of articles focused on IRT, and this may have impacted the distribution of the most commonly observed disease-causing and -associated genetic variants as well. Studies which focused on a single genetic disorder, such as *NFKB1*, were excluded from collation within our SLR, and this may have introduced some bias within the collated frequencies of immune dysregulation complications among patients with CVIDc given that *NFKB1* is a common cause of CVID. However, at least one large study of more than 100 individuals with pathogenic variants in *NFKB1* reported similar rates of autoimmunity (57.4%), lymphoproliferation (52.4%), noninfectious enteropathy (23.1%), and autoinflammation (29.6%) as summarized here for patients with CVIDc (107).

An additional limitation is the lack of ability to formally compare the frequencies of the various noninfectious manifestations in patients with CVIDc to the general population. While it is evident that manifestations such as splenomegaly, ILD, and enteropathy are complications driven by immune dysregulation in patients with CVIDc, the observed frequencies of medical diseases that are common in the general population such as endocrinopathies are difficult to interpret. Type 1 diabetes, for example, was observed at a median frequency of 2.2% among the included studies but has a similar self-reported prevalence of 0.53% among adults in the United States (114). Hypothyroidism was observed at a median frequency of 6.9%, while estimates of the prevalence of hypothyroidism in the general U.S. population are 10–12% (115). Rates of endocrine disorders in patients with CVID may simply reflect those of the general population. Additionally, diagnoses within other organ systems such as asthma are challenging to fully interpret. The median frequency of asthma observed among included studies was 30.5% of patients with CVID, which is ∼4 times higher than the estimated prevalence of asthma among U.S. adults of 8% (116). Data collected from 1,470 patients with CVID by the United States Immunodeficiency Network (USIDNET) similarly suggest that at least 40% of patients with CVID have a diagnosis of asthma (117). However, asthma may be over diagnosed in patients with CVID due to other comorbidities, and asthma diagnoses are further complicated by inaccurate diagnoses in even the general population where up to one in three patients diagnosed with asthma may not be able to have their diagnosis confirmed (118). Accurate direct comparisons of the rates of common diseases would require well-matched cohorts based on demographics, such as age, sex, race and ethnicity, poverty level, and other factors, which may affect risks of specific medical diseases. Future efforts that focus on the formal comparison of the rates of complications for which screening examinations are available may improve patient care by providing data upon which to develop screening recommendations for patients with CVIDc.

Despite these limitations, the collated data from this SLR provide a timely detailed summary of the most frequently reported noninfectious clinical manifestations, disease-causing or -associated genetic variants, immunosuppressive and immunomodulatory treatments, and survival outcomes among patients with CVIDc. To the best of our knowledge, this is the first SLR of its kind as it reports the observed CVID clinical profile with an emphasis on noninfectious, nonmalignant clinical manifestations of immune dysregulation, which drive almost all the observed mortality in patients with CVID (44). The CVID SLRs published prior to the current SLR described infectious manifestations (119) or focused on a specific manifestation associated with CVID (e.g., CVID and connective tissue disorders) (120). A review of the underlying genetic associations with organ-specific immunopathologies observed in pediatric CVID was also recently published but focused on potential mechanisms (121). In contrast, our quantitative findings emphasize the general pervasiveness of lymphoproliferative, autoimmune, and inflammatory manifestations of immune dysregulation in the wider CVID population and their negative impacts on disease burden and mortality. This SLR highlights an urgent and unmet need to develop novel treatments to target the manifestations of immune dysregulation in patients with CVIDc with the goal of improving overall outcomes.

## Materials and methods

### Study design

A comprehensive SLR was conducted to characterize the clinical profile of patients with CVID and noninfectious clinical manifestations. The study aimed to summarize the frequency of noninfectious manifestations, genetic causes, age of disease onset and diagnosis, treatment landscape, and survival outcomes in patients with CVID. The SLR was conducted in accordance with the PRISMA guidelines (122).

### Eligibility criteria

Study eligibility criteria were pre-specified in terms of the PICOS structure outlined in Table 1, which guided the identification and selection of studies relevant to the SLR.

The population of interest consisted of humans with CVID; animal studies and *in vitro* studies were excluded. The primary studies of interest were those that reported the noninfectious clinical manifestations of large cohorts of patients with CVID (i.e., with sample sizes >10 patients) without focusing on one specific genetic cause. A secondary consideration was studies that reported treatment responses and outcomes in patients with CVID, regardless of cohort size. Studies focusing on vaccine responses, IRT, and COVID-19 were excluded. In terms of study design, clinical trials and observational studies were of highest interest. Editorials, narrative reviews, letters, and notes were excluded; case reports and case series were also excluded unless they reported treatment responses and outcomes. Conference abstracts captured via the main database search were excluded as relevant proceedings of target conferences were searched separately. Only studies published in English were of interest; no time restriction was applied.

### Data sources and search strategy

Relevant studies were identified using the Ovid platform to conduct comprehensive literature searches of the Embase, MEDLINE, and CENTRAL databases. The searches were executed on April 15, 2024, using predefined search strategies (Table S5) and included a combination of free-text terms and indexed medical subject heading terms specific to each database as recommended by the Cochrane Collaboration (123). In addition, conference proceedings from the Clinical Immunology Society, European Society for Immunodeficiencies, American Society of Hematology, and American Academy of Allergy, Asthma & Immunology held between 2022 and 2024 were searched through the Northern Light Life Sciences Conference Abstracts database on Ovid to identify relevant abstracts.

### Study selection and data extraction

The resulting titles, abstracts, and full-text publications were screened by a single reviewer, and a second reviewer screened ∼25% of excluded abstracts for quality assurance. Discrepancies were resolved by mutual consensus to reach a final decision.

Once the data extraction template was finalized, one reviewer extracted data on study characteristics, baseline characteristics of the target population, intervention characteristics (when reported), and outcomes of interest for the final list of included studies. A second reviewer conducted a final check of all extracted data to ensure consistency in reporting information across publications.

### Outcomes

The clinical profiles reported herein are presented as a calculated median of the percentages of patients with each specific feature (i.e., genetics, clinical manifestations, causes of death, and treatments used) across all applicable studies. For each study, percentages were either directly provided or they were calculated manually based on the number of patients with a specific feature out of the total number of patients in the respective cohort. For the summary of genetic observations, 1 study included only benign variants or VUSs and was therefore excluded from the genetic analysis (28).

Noninfectious, nonmalignant clinical manifestations of immune dysregulation are presented by organ system or by subspecialty area. Manifestations reported by at least 10 articles are included in the figures, and data are presented as a calculated median percentage of patients with each manifestation across all respective studies. Three studies reported clinical information on >1 CVID cohort (12, 15, 57). These studies may have more than 1 value contributing to the combined median for each parameter reported but are counted as 1 study. Furthermore, manifestations were combined into different categories as shown in Fig. 3 through Fig. 7; as such, some studies may have multiple values entered per category. For example, 1 study reported the percentage of patients with Crohn’s disease and those with ulcerative colitis as separate values; however, herein both values were reported under the IBD category in Fig. 4. The specific publications reporting each manifestation or clinical parameter are detailed in Table S3.

### Online supplemental material

Supplementary files include Tables S1–S5. Table S1 reports the reasoning for exclusion of each individual publication during the full-text screening process. Table S2 provides a comprehensive list of publications reporting specific study characteristics or patient demographics. Table S3 provides the full list of publications reporting specific noninfectious manifestations, treatments, and mortality information. Table S4 reports the HRs, P values, type of analysis, and the respective publication for each specific manifestation. Table S5 provides the search strategy used to conduct the SLR.

## Supplementary Material

Table S1 shows reasoning for publication exclusions during full-text screening.

Table S2 shows publications reporting specific study characteristics or patient demographics.

Table S3 shows publications reporting noninfectious manifestations, treatments, and mortality.

Table S4 shows all manifestations with mortality HRs and P values.

Table S5 shows search strategy.

## Data Availability

The search strategy used to obtain the data included in the current SLR is available in Table S5. The data included for each clinical feature are available in the respective publications referenced in Tables S2, S3, and S4.
