# Supplementary material for: A systematic literature review of CVID reveals pervasive detrimental noninfectious manifestations
Source: J Hum Immun. 2025 Oct 24;2(1):e20250157. doi: 10.70962/jhi.20250157 (PMC13177467; doi:10.70962/jhi.20250157)
Supplement: Table S1 — shows reasoning for publication exclusions during full-text screening. [file jhi_20250157_tables1.docx]

**Supplementary files:**

**Table S1:** Reasoning for publication exclusions during full text screening

| Author | Year | Title | Reason for exclusion |
| --- | --- | --- | --- |
| Abati et al | 2018 | Central nervous system involvement in common variable immunodeficiency: A case of acute unilateral optic neuritis in a 26 -year-old italian patient | Case study/report |
| Abdelmoumen et al | 2023 | Jak inhibition with baricitinib for severe cvid-related enteropathy: A case report | Case study/report |
| Abdollahzade et al | 2011 | The clinical immunological and long-term follow-up of pediatric patients with common variable immunodeficiency | Not in English |
| Abolhassani et al | 2013 | Expression of activation-induced cytidine deaminase gene in b lymphocytes of patients with common variable immunodeficiency | Study design/outcomes reported |
| Abyazi et al | 2022 | Convergence of cytokine dysregulation and antibody deficiency in common variable immunodeficiency with inflammatory complications | Study design/outcomes reported |
| Adin-Cinar et al | 2021 | Functions of nk and inkt cells in pediatric and adult cvid, ataxia telangiectasia and agammaglobulinemia patients | Low N/cohort characteristics |
| Aghamohammadi et al | 2010 | Comparison of pulmonary diseases in common variable immunodeficiency and x-linked agammaglobulinaemia | Study design/outcomes reported |
| Aghamohammadi et al | 2005 | Clinical and immunological features of 65 iranian patients with common variable immunodeficiency | Study design/outcomes reported |
| Aghamohammadi et al | 2009 | Infectious and non-infectious complications among undiagnosed patients with common variable immunodeficiency | Study design/outcomes reported |
| Agondi et al | 2013 | Can patients with common variable immunodeficiency have allergic rhinitis? | Study design/outcomes reported |
| Akazawa et al | 2014 | Infliximab therapy for crohn's-like disease in common variable immunodeficiency complicated by massive intestinal hemorrhage: A case report | Case study/report |
| Al Kindi et al | 2012 | Utility of peripheral blood b cell subsets analysis in common variable immunodeficiency | Study design/outcomes reported |
| Al-Ahmad et al | 2010 | Successful use of rituximab in refractory idiopathic thrombocytopenic purpura in a patient with common variable immunodeficiency | IRT/ treatment not relevant |
| Albert et al | 2022 | Hematopoietic stem cell transplantation for adolescents and adults with inborn errors of immunity: An ebmt iewp study | Study design/outcomes reported |
| Albshesh et al | 2022 | Primary hypogammaglobulinaemia with inflammatory bowel disease-like features: An ecco confer multicentre case series | Low N/cohort characteristics |
| Almeida et al | 2011 | Correlation between the clinical and immunological phenotypes in adults and children with common variable immunodeficiency | Not in English |
| Almejun et al | 2012 | Immunological characteristics and two novel mutations in taci in a cohort of 28 pediatric patients with common variable immunodeficiency | Genetic CVID only |
| Alper et al | 2000 | Prospective analysis suggests susceptibility genes for deficiencies of iga and several other immunoglobulins on the [hla-b8, sc01, dr3] conserved extended haplotype | Not relevant |
| Amanzadeh et al | 2012 | Association of hla-drb1, dqa1 and dqb1 alleles and haplotypes with common variable immunodeficiency in iranian patients | Low N/cohort characteristics |
| Ambrus Jr et al | 1991 | Improved in vitro antigen-specific antibody synthesis in two patients with common variable immunodeficiency taking an oral cyclooxygenase and lipoxygenase inhibitor (ketoprofen) | Low N/cohort characteristics |
| Amer et al | 2007 | Resolution of juvenile idiopathic arthritis-associated uveitis after development of common variable immunodeficiency | IRT/ treatment not relevant |
| Amo Alonso et al | 2017 | Infliximab as crohn's disease treatment in a patient with a history of common variable immunodeficiency | Not in English |
| Angarola et al | 2024 | Secondary hypogammaglobulinemia in adults-a large retrospective cohort study | Low N/cohort characteristics |
| Arandi et al | 2013 | Frequency and expression of inhibitory markers of cd4<sup>+</sup>cd25<sup>+</sup>foxp3<sup>+</sup> regulatory t cells in patients with common variable immunodeficiency | Not relevant |
| Ardeniz et al | 2010 | Clinical and immunological analysis of 23 adult patients with common variable immunodeficiency | Low N/cohort characteristics |
| Ardeniz and Cunningham-Rundles | 2009 | Granulomatous disease in common variable immunodeficiency | IRT/ treatment not relevant |
| Arish et al | 2006 | Lymphocytic interstitial pneumonia associated with common variable immunodeficiency resolved with intravenous immunoglobulins | Case study/report |
| Aspalter et al | 2000 | Deficiency in circulating natural killer (nk) cell subsets in common variable immunodeficiency and x-linked agammaglobulinaemia | Study design/outcomes reported |
| Atschekzei et al | 2016 | Limitation of simultaneous analysis of t-cell receptor and kappa-deleting recombination excision circles based on multiplex real-time polymerase chain reaction in common variable immunodeficiency patients | Study design/outcomes reported |
| Aukrust et al | 1992 | Raised serum neopterin levels in patients with primary hypogammaglobulinaemia; correlation to other immunological parameters and to clinical and histological features | Low N/cohort characteristics |
| Aukrust et al | 1994 | Elevated serum levels of interleukin-4 and interleukin-6 in patients with common variable immunodeficiency (cvi) are associated with chronic immune activation and low numbers of cd4+ lymphocytes | Low N/cohort characteristics |
| Aukrust et al | 2000 | Decreased vitamin a levels in common variable immunodeficiency: Vitamin a supplementation in vivo enhances immunoglobulin production and downregulates inflammatory responses | Low N/cohort characteristics |
| Aydogan et al | 2008 | Clinical and immunologic features of pediatric patients with common variable immunodeficiency and respiratory complications | Low N/cohort characteristics |
| Aytekin et al | 2021 | Nephrological factors may cause kidney dysfunction in patients with common variable immunodeficiency | Low N/cohort characteristics |
| Aytekin et al | 2019 | Risk factors of bronchiectasis in adult patients with common variable immunodeficiency | Not relevant |
| Azarsiz et al | 2017 | Chitotriosidase enzyme activity: Is this a possible chronic inflammation marker in children with common variable immunodeficiency and early atherosclerosis? | Low N/cohort characteristics |
| Azizi et al | 2018 | Rheumatologic complications in a cohort of 227 patients with common variable immunodeficiency | Not relevant |
| Azizi et al | 2017 | Autoimmunity in a cohort of 471 patients with primary antibody deficiencies | Low N/cohort characteristics |
| Babaie et al | 2017 | Surveillance of primary immunodeficiency disorders in mofid children's hospital: A 10-year retrospective experience | Low N/cohort characteristics |
| Ballegaard et al | 2013 | Long-term follow-up on affinity maturation and memory b-cell generation in patients with common variable immunodeficiency | Not relevant |
| Banday et al | 2020 | Refractory autoimmune cytopenia in a young boy with a novel lrba mutation successfully managed with sirolimus | IRT/ treatment not relevant |
| Banh et al | 2012 | Persistent elevation of peripheral blood myeloid cell counts associated with omalizumab therapy | IRT/ treatment not relevant |
| Barbosa et al | 2014 | Reduced baff-r and increased taci expression in common variable immunodeficiency | Study design/outcomes reported |
| Baris et al | 2011 | Efficacy of intravenous immunoglobulin treatment in children with common variable immunodeficiency | IRT/ treatment not relevant |
| Barmettler et al | 2020 | Gastrointestinal manifestations in common variable immunodeficiency (cvid) are associated with an altered immunophenotype including b- and t-cell dysregulation | Study design/outcomes reported |
| Barmettler and Price | 2015 | Continuing igg replacement therapy for hypogammaglobulinemia after rituximab-for how long? | Not relevant |
| Bayrakci et al | 2005 | The efficacy of immunoglobulin replacement therapy in the long-term follow-up of the b-cell deficiencies (xla, him, cvid) | Low N/cohort characteristics |
| Bayraktaroglu et al | 2023 | Comparison of chest computed tomography and 3-t magnetic resonance imaging results in patients with common variable immunodeficiency | Low N/cohort characteristics |
| Bayrhuber et al | 2019 | Perceived health of patients with common variable immunodeficiency - a cluster analysis | Study design/outcomes reported |
| Ben Khaled et al | 2023 | Clinical features and predictors of osteoarticular manifestations in common variable immunodeficiency | Low N/cohort characteristics |
| Berbers et al | 2021 | Targeted proteomics reveals inflammatory pathways that classify immune dysregulation in common variable immunodeficiency | Not relevant |
| Berbers et al | 2020 | Low iga associated with oropharyngeal microbiota changes and lung disease in primary antibody deficiency | Not relevant |
| Berbers et al | 2021 | Chronically activated t-cells retain their inflammatory properties in common variable immunodeficiency | Study design/outcomes reported |
| Berron-Ruiz et al | 2016 | Impaired selective cytokine production by cd4(+) t cells in common variable immunodeficiency associated with the absence of memory b cells | Low N/cohort characteristics |
| Bhattad et al | 2023 | Profile of 208 patients with inborn errors of immunity at a tertiary care center in south india | Low N/cohort characteristics |
| Billich | 2007 | Drug evaluation: Apilimod, an oral il-12/il-23 inhibitor for the treatment of autoimmune diseases and common variable immunodeficiency | Study design/outcomes reported |
| Bintalib et al | 2022 | Corticosteroid-induced remission and mycophenolate maintenance therapy in granulomatous lymphocytic interstitial lung disease: Long-term, longitudinal change in lung function in a single-centre cohort | Low N/cohort characteristics |
| Bisgin et al | 2021 | The impact of rare and low-frequency genetic variants in common variable immunodeficiency (cvid) | Study design/outcomes reported |
| Blazek et al | 2015 | Human immunoglobulin (kiovig<sup></sup>/gammagard liquid<sup></sup>) for immunodeficiency and autoimmune diseases: An observational cohort study | Low N/cohort characteristics |
| Bogaert et al | 2017 | The immunophenotypic fingerprint of patients with primary antibody deficiencies is partially present in their asymptomatic first-degree relatives | Study design/outcomes reported |
| Boloursaz et al | 2012 | Chest ct manifestations in children with cvid: A 10-year report | Low N/cohort characteristics |
| Bondioni et al | 2007 | Pulmonary and sinusal changes in 45 patients with primary immunodeficiencies: Computed tomography evaluation | Study design/outcomes reported |
| Bondioni et al | 2010 | Common variable immunodeficiency: Computed tomography evaluation of bronchopulmonary changes including nodular lesions in 40 patients. Correlation with clinical and immunological data | Low N/cohort characteristics |
| Bonhomme et al | 2000 | Impaired antibody affinity maturation process characterizes a subset of patients with common variable immunodeficiency | Not relevant |
| Borzutzky et al | 2018 | Defective tlr9-driven stat3 activation in b cells of patients with cvid | Low N/cohort characteristics |
| Botelho Alves et al | 2024 | Expression of il-17ra in innate cells of patients with common variable immunodeficiency (cvid) and its clinical implications | Low N/cohort characteristics |
| Boumaza et al | 2023 | Infectious risk when prescribing rituximab in patients with hypogammaglobulinemia acquired in the setting of autoimmune diseases | Low N/cohort characteristics |
| Bouvry et al | 2013 | Granulomatosis-associated common variable immunodeficiency disorder: A case - control study versus sarcoidosis | Low N/cohort characteristics |
| Bright et al | 2013 | Changes in b cell immunophenotype in common variable immunodeficiency: Cause or effect - is bronchiectasis indicative of undiagnosed immunodeficiency? | Not relevant |
| Bruns et al | 2022 | Common variable immunodeficiency-associated cancers: The role of clinical phenotypes, immunological and genetic factors | Study design/outcomes reported |
| Busse et al | 2002 | Efficacy of intravenous immunoglobulin in the prevention of pneumonia in patients with common variable immunodeficiency | IRT/ treatment not relevant |
| Caldirola et al | 2020 | Immune monitoring of patients with primary immune regulation disorders unravels higher frequencies of follicular t cells with different profiles that associate with alterations in b cell subsets | Low N/cohort characteristics |
| Calheiro dos Santos-Valente et al | 2012 | Assessment of nutritional status: Vitamin a and zinc in patients with common variable immunodeficiency | Low N/cohort characteristics |
| Caliskaner et al | 2016 | Common variable immunodeficiency in adults requires reserved protocols for long-term follow-up | Low N/cohort characteristics |
| Camacho-Ordonez et al | 2022 | Integrated multi-omics analyses of nfkb1 patients b cells points towards an up regulation of nf-kappab network inhibitors | Low N/cohort characteristics |
| Capistrano et al | 2018 | Renal evaluation in common variable immunodeficiency | Low N/cohort characteristics |
| Carsetti et al | 2020 | Lack of gut secretory immunoglobulin a in memory b-cell dysfunction-associated disorders: A possible gut-spleen axis | Low N/cohort characteristics |
| Carsetti et al | 2005 | The loss of igm memory b cells correlates with clinical disease in common variable immunodeficiency | Low N/cohort characteristics |
| Carter et al | 2013 | Cvid patients with autoimmunity have elevated t cell expression of granzyme b and hla-dr and reduced levels of treg cells | Not relevant |
| Carvalho et al | 2010 | Skewed distribution of circulating activated natural killer t (nkt) cells in patients with common variable immunodeficiency disorders (cvid) | Low N/cohort characteristics |
| Cavaliere et al | 2015 | Intravenous immunoglobulin replacement induces an in vivo reduction of inflammatory monocytes and retains the monocyte ability to respond to bacterial stimulation in patients with common variable immunodeficiencies | Not relevant |
| Caza et al | 2020 | Renal manifestations of common variable immunodeficiency | Low N/cohort characteristics |
| Chakraborty et al | 2023 | Interleukin-9 rescues class switching of memory b cells derived from common variable immunodeficiency patients | Low N/cohort characteristics |
| Chen et al | 2023 | Endoscopic and histopathological hints on infections in patients of common variable immunodeficiency disorder with gastrointestinal symptoms | Low N/cohort characteristics |
| Christiansen et al | 2019 | Identification of novel genetic variants in cvid patients with autoimmunity, autoinflammation, or malignancy | Low N/cohort characteristics |
| Clarsund et al | 2016 | Evaluation of coldzyme mouth spray on prevention of upper respiratory tract infections in a boy with primary immunodeficiency: A case report | Case study/report |
| Comberiati et al | 2019 | Refractory chronic spontaneous urticaria treated with omalizumab in an adolescent with common variable immunodeficiency | Not relevant |
| Conti et al | 2022 | Refractory immune thrombocytopenia successfully treated with bortezomib in a child with 22q11.2 deletion syndrome, complicated by evans syndrome and hypogammaglobulinemia | Study design/outcomes reported |
| Coraglia et al | 2016 | Common variable immunodeficiency and circulating tfh | Low N/cohort characteristics |
| Correa-Jimenez et al | 2023 | Respiratory comorbidities associated with bronchiectasis in patients with common variable immunodeficiency in the usidnet registry | Not relevant |
| Crotty et al | 2020 | Spectrum of hepatic manifestations of common variable immunodeficiency | Study design/outcomes reported |
| Cunningham-Rundles et al | 2002 | Lymphomas of mucosal-associated lymphoid tissue in common variable immunodeficiency | Low N/cohort characteristics |
| Cunningham-Rundles et al | 1994 | Brief report: Enhanced humoral immunity in common variable immunodeficiency after long-term treatment with polyethylene glycol- conjugated interleukin-2 | Case study/report |
| Curtin et al | 1995 | Mediastinal lymph node enlargement and splenomegaly in primary hypogammaglobulinaemia | Study design/outcomes reported |
| Danieli et al | 2022 | Common variable immunodeficiency in elderly patients: A long-term clinical experience | Not relevant |
| Danieli et al | 2022 | Replacement and immunomodulatory activities of 20% subcutaneous immunoglobulin treatment: A single-center retrospective study in autoimmune myositis and cvid patients | Low N/cohort characteristics |
| Daniels et al | 2009 | Hepatitis in common variable immunodeficiency | Low N/cohort characteristics |
| Dasanayake et al | 2023 | Inborn errors of immunity-the sri lankan experience 2010-2022 | Low N/cohort characteristics |
| De Gracia et al | 2004 | Immunoglobulin therapy to control lung damage in patients with common variable immunodeficiency | Not relevant |
| De La Concha et al | 1999 | Hla class ii homozygosity confers susceptibility to common variable immunodeficiency (cvid) | Genetic CVID only |
| de Lollo et al | 2016 | Impaired cd8(+) t cell responses upon toll-like receptor activation in common variable immunodeficiency | Low N/cohort characteristics |
| de Morales et al | 2017 | Successful treatment of common variable immunodeficiency-associated inflammatory bowel disease with ustekinumab | Not relevant |
| de Valles-Ibanez et al | 2018 | Evaluating the genetics of common variable immunodeficiency: Monogenetic model and beyond | Study design/outcomes reported |
| De Vera et al | 2004 | Assessing thymopoiesis in patients with common variable immunodeficiency as measured by t-cell receptor excision circles | Low N/cohort characteristics |
| Del Pino-Molina et al | 2020 | Dissection of the pre-germinal center b-cell maturation pathway in common variable immunodeficiency based on standardized flow cytometric euroflow tools | Not relevant |
| Della Bella et al | 1997 | Successful treatment of common variable immunodeficiency and related disorders with cimetidine and zinc sulfate | Study design/outcomes reported |
| Detkova et al | 2007 | Common variable immunodeficiency: Association between memory b cells and lung diseases | Not relevant |
| Diaz-Alberola et al | 2022 | Incidence, management experience and characteristics of patients with giardiasis and common variable immunodeficiency | Study design/outcomes reported |
| DiGiacomo et al | 2023 | Predominant antibody deficiency and risk of microscopic colitis: A nationwide case-control study in sweden | Not relevant |
| DiGiacomo et al | 2022 | Liver stiffness by transient elastography correlates with degree of portal hypertension in common variable immunodeficiency patients with nodular regenerative hyperplasia | Study design/outcomes reported |
| Dong et al | 2016 | Adult common variable immunodeficiency | Low N/cohort characteristics |
| Driessen et al | 2011 | B-cell replication history and somatic hypermutation status identify distinct pathophysiologic backgrounds in common variable immunodeficiency | Not relevant |
| Duvvuri et al | 2011 | Altered spectrum of somatic hypermutation in common variable immunodeficiency disease characteristic of defective repair of mutations | Low N/cohort characteristics |
| Ebbo et al | 2016 | Low circulating natural killer cell counts are associated with severe disease in patients with common variable immunodeficiency | Not relevant |
| Edwards et al | 2019 | Predominantly antibody-deficient patients with non-infectious complications have reduced naive b, treg, th17, and tfh17 cells | Low N/cohort characteristics |
| El-Shanawany et al | 2007 | Response of refractory immune thrombocytopenic purpura in a patient with common variable immunodeficiency to treatment with rituximab | Low N/cohort characteristics |
| Emerson et al | 2021 | Duodenal plasma cells correspond to serum iga in common variable immunodeficiency | Not relevant |
| Engstrom et al | 1992 | Oral conditions in individuals with selective immunoglobulin a deficiency and common variable immunodeficiency | Study design/outcomes reported |
| Erdem et al | 2019 | Characteristics of the patients followed with the diagnosis of common variable immunodeficiency and the complications | Low N/cohort characteristics |
| Esenboga et al | 2021 | Respiratory system findings in pediatric patients with primary immunodeficiency | Low N/cohort characteristics |
| Eskandarian et al | 2019 | Assessing the functional relevance of variants in the ikaros family zinc finger protein 1 (ikzf1) in a cohort of patients with primary immunodeficiency | Low N/cohort characteristics |
| Farber et al | 1994 | Tumor necrosis factor and intravenous gammaglobulins in common variable immunodeficiency | Low N/cohort characteristics |
| Farrokhi et al | 2020 | Increased expression of b lymphocyte induced maturation protein 1 (blimp1) in patients with common variable immunodeficiency (cvid) | Low N/cohort characteristics |
| Fernandes et al | 2016 | Salivary immunoglobulins in individuals with common variable immunodeficiency | Not relevant |
| Fernandez Romero et al | 2013 | Common variable immunodeficiency. Epidemiology and clinical manifestations in 69 patients | Not in English |
| Fiedorova et al | 2019 | Bacterial but not fungal gut microbiota alterations are associated with common variable immunodeficiency (cvid) phenotype | Low N/cohort characteristics |
| Firtina et al | 2022 | Primary antibody deficiencies in turkey: Molecular and clinical aspects | Not relevant |
| Fischer et al | 2017 | Autoimmune and inflammatory manifestations occur frequently in patients with primary immunodeficiencies | Study design/outcomes reported |
| Friedmann et al | 2020 | Bronchoalveolar lavage fluid reflects a t<sub>h</sub>1-cd21<sup>low</sup> b-cell interaction in cvid-related interstitial lung disease | Not relevant |
| Fukushima et al | 2008 | A case of severe recurrent hepatitis with common variable immunodeficiency | Case study/report |
| Fulcher et al | 2009 | Invariant natural killer (ink) t cell deficiency in patients with common variable immunodeficiency | Study design/outcomes reported |
| Fuss et al | 2013 | Nodular regenerative hyperplasia in common variable immunodeficiency | Low N/cohort characteristics |
| G et al | 2020 | Bone metabolism alterations in patients with common variable immune deficiency: A retrospective cohort study | Duplicate |
| Gao et al | 2013 | Common variable immunodeficiency is associated with a functional deficiency of invariant natural killer t cells | Not relevant |
| Geier et al | 2017 | Reduced numbers of circulating group 2 innate lymphoid cells in patients with common variable immunodeficiency | Not relevant |
| Gill and Betschel | 2018 | Timing of infections in patients with primary immunodeficiencies treated with intravenous immunoglobulin (ivig) | Low N/cohort characteristics |
| Giovannetti et al | 2007 | Unravelling the complexity of t cell abnormalities in common variable immunodeficiency | Study design/outcomes reported |
| Giraldo-Ocampo et al | 2022 | B cell subsets in colombian adults with predominantly antibody deficiencies, bronchiectasis or recurrent pneumonia | Low N/cohort characteristics |
| Globig et al | 2021 | International multicenter experience of transjugular intrahepatic portosystemic shunt implantation in patients with common variable immunodeficiency | Low N/cohort characteristics |
| Globig et al | 2022 | Evaluation of laboratory and sonographic parameters for detection of portal hypertension in patients with common variable immunodeficiency | Low N/cohort characteristics |
| Gompels et al | 2003 | Lymphoproliferative disease in antibody deficiency: A multi-centre study | Low N/cohort characteristics |
| Goussault et al | 2019 | Primary immunodeficiency-related bronchiectasis in adults: Comparison with bronchiectasis of other etiologies in a french reference center | Study design/outcomes reported |
| Gregersen et al | 2010 | Development of pulmonary abnormalities in patients with common variable immunodeficiency: Associations with clinical and immunologic factors | Not relevant |
| Gregersen et al | 2009 | High resolution computed tomography and pulmonary function in common variable immunodeficiency | Not relevant |
| Gregersen et al | 2013 | Lung disease, t-cells and inflammation in common variable immunodeficiency disorders | Low N/cohort characteristics |
| Grzesk et al | 2021 | Common variable immunodeficiency: Different faces of the same disease | Low N/cohort characteristics |
| Gualdi et al | 2015 | Burden of skin disease in selective iga deficiency and common variable immunodeficiency | Case study/report |
| Guazzi et al | 2002 | Assessment of thymic output in common variable immunodeficiency patients by evaluation of t cell receptor excision circles | Low N/cohort characteristics |
| Guevara-Hoyer et al | 2021 | Variable immunodeficiency score upfront analytical link (visual), a proposal for combined prognostic score at diagnosis of common variable immunodeficiency | Not relevant |
| Guevara-Hoyer et al | 2020 | Variable immunodeficiency study: Evaluation of two european cohorts within a variety of clinical phenotypes | Not relevant |
| Guffroy et al | 2017 | Neutropenia in patients with common variable immunodeficiency: A rare event associated with severe outcome | Low N/cohort characteristics |
| Gullo et al | 2020 | The dysfunctional immune system in common variable immunodeficiency increases the susceptibility to gastric cancer | Low N/cohort characteristics |
| Gupta et al | 2019 | Primary immunodeficiency disorders among north indian children | Low N/cohort characteristics |
| Gutierrez et al | 2018 | Phenotypic characterization of patients with rheumatologic manifestations of common variable immunodeficiency | Not relevant |
| Hartono et al | 2017 | Predictors of granulomatous lymphocytic interstitial lung disease in common variable immunodeficiency | Low N/cohort characteristics |
| Hermaszewski and Webster | 1993 | Primary hypogammaglobulinaemia: A survey of clinical manifestations and complications | Not relevant |
| Horn et al | 2009 | Decrease in phenotypic regulatory t cells in subsets of patients with common variable immunodeficiency | Not relevant |
| Hultberg et al | 2023 | In-depth immune profiling reveals advanced b- and t-cell differentiation to be associated with th1-driven immune dysregulation in common variable immunodeficiency | Low N/cohort characteristics |
| Hwangpo et al | 2020 | Use of fef25-75% to guide igg dosing to protect pulmonary function in cvid | Not relevant |
| Ilkjaer et al | 2023 | Evaluating drug prescription patterns in undiagnosed common variable immunodeficiency patients | Study design/outcomes reported |
| Ilkjaer et al | 2019 | How to identify common variable immunodeficiency patients earlier: General practice patterns | Study design/outcomes reported |
| Janssen et al | 2017 | Igg trough levels and progression of pulmonary disease in pediatric and adult common variable immunodeficiency disorder patients | Not relevant |
| Johnson et al | 2022 | Electronic health record signatures identify undiagnosed patients with common variable immunodeficiency disease | Study design/outcomes reported |
| Johnston et al | 2004 | Echocardiographic abnormalities in primary antibody deficiency | Low N/cohort characteristics |
| Jolles et al | 2017 | Screening protocols to monitor respiratory status in primary immunodeficiency disease: Findings from a european survey and subclinical infection working group | Study design/outcomes reported |
| Jorgensen et al | 2024 | Retraction note: Rifaximin alters gut microbiota profile, but does not affect systemic inflammation - a randomized controlled trial in common variable immunodeficiency | Retraction |
| Kainulainen et al | 1999 | Pulmonary abnormalities in patients with primary hypogammaglobulinemia | Low N/cohort characteristics |
| Kaplan et al | 2014 | Rituximab and immune deficiency: Case series and review of the literature | Low N/cohort characteristics |
| Karali et al | 2020 | Evaluation of pulmonary findings in patients with humoral immunodeficiency | Low N/cohort characteristics |
| Karam et al | 2013 | Pulmonary and sinus imaging in common variable immunodeficiency: What do we expect to find? | Study design/outcomes reported |
| Kellner et al | 2019 | Cellular defects in cvid patients with chronic lung disease in the usidnet registry | Not relevant |
| Kilinc et al | 2023 | Relationship between autoimmune diseases and serum basal immunoglobulin e levels in patients with common variable immunodeficiency | Not relevant |
| Kinlen et al | 1985 | Prospective study of cancer in patients with hypogammaglobulinaemia | Not relevant |
| Kofod-Olsen et al | 2016 | Altered fraction of regulatory b and t cells is correlated with autoimmune phenomena and splenomegaly in patients with cvid | Not relevant |
| Kralickova et al | 2018 | Cvid-associated tumors: Czech nationwide study focused on epidemiology, immunology, and genetic background in a cohort of patients with cvid | Study design/outcomes reported |
| Kutukculer et al | 2016 | Cd4<sup>+</sup>cd25<sup>+</sup>foxp3<sup>+</sup> t regulatory cells, th1 (ccr5, il-2, ifn-gamma) and th2 (ccr4, il-4, il-13) type chemokine receptors and intracellular cytokines in children with common variable immunodeficiency | Low N/cohort characteristics |
| Kutukculer and Gulez | 2009 | The outcome of patients with unclassified hypogammaglobulinemia in early childhood | Low N/cohort characteristics |
| Lanio et al | 2009 | Immunophenotypic profile of t cells in common variable immunodeficiency: Is there an association with different clinical findings? | Low N/cohort characteristics |
| Larsen et al | 2020 | Glild revisited: Pulmonary pathology of common variable and selective iga immunodeficiency | Study design/outcomes reported |
| Lima et al | 2022 | Liver disease accompanied by enteropathy in common variable immunodeficiency: Common pathophysiological mechanisms | Not relevant |
| Lin et al | 2015 | Clinical and immunological features of common variable immunodeficiency in china | Not relevant |
| Liu et al | 2023 | Genetic characteristics of common variable immunodeficiency patients with autoimmunity | Low N/cohort characteristics |
| Lopez et al | 2020 | Lung disease in patients with common variable immunodeficiency | Not relevant |
| Lougaris et al | 2016 | Correlation of bone marrow abnormalities, peripheral lymphocyte subsets and clinical features in uncomplicated common variable immunodeficiency (cvid) patients | Low N/cohort characteristics |
| Lougaris et al | 2015 | Gastrointestinal pathologic abnormalities in pediatric- and adult-onset common variable immunodeficiency | Low N/cohort characteristics |
| Luzi et al | 2003 | Duodenal pathology and clinical-immunological implications in common variable immunodeficiency patients | Not relevant |
| Maarschalk-Ellerbroek et al | 2013 | Outcome of screening endoscopy in common variable immunodeficiency disorder and x-linked agammaglobulinemia | Not relevant |
| Maglione et al | 2015 | Progression of common variable immunodeficiency interstitial lung disease accompanies distinct pulmonary and laboratory findings | Low N/cohort characteristics |
| Malamut et al | 2010 | The enteropathy associated with common variable immunodeficiency: The delineated frontiers with celiac disease | Not relevant |
| Marschall et al | 2015 | The swiss national registry for primary immunodeficiencies: Report on the first 6 years' activity from 2008 to 2014 | Study design/outcomes reported |
| Martinez Garcia et al | 2001 | Respiratory disorders in common variable immunodeficiency | Low N/cohort characteristics |
| Mechanic et al | 1997 | Granulomatous disease in common variable immunodeficiency | Low N/cohort characteristics |
| Milota et al | 2019 | Bronchial asthma and bronchial hyperresponsiveness and their characteristics in patients with common variable immunodeficiency | Low N/cohort characteristics |
| Mirzaee et al | 2018 | Registry of clinical data and laboratory findings in 80 patients with primary immunodeficiencies in mofid children hospital | Low N/cohort characteristics |
| Moazzami et al | 2020 | Comprehensive assessment of respiratory complications in patients with common variable immunodeficiency | Not relevant |
| Mohebbi et al | 2017 | Comparison of bone mineral density in common variable immunodeficiency and x-linked agammaglobulinaemia patients | Not relevant |
| Moratto et al | 2006 | Combined decrease of defined b and t cell subsets in a group of common variable immunodeficiency patients | Low N/cohort characteristics |
| Mouillot et al | 2010 | B-cell and t-cell phenotypes in cvid patients correlate with the clinical phenotype of the disease | Not relevant |
| Musabak and Erdogan | 2021 | Clinical features and immunoglobulin replacement therapy outcomes of adults with common variable immunodeficiency: A single centre experience | Low N/cohort characteristics |
| Ochtrop et al | 2011 | T and b lymphocyte abnormalities in bone marrow biopsies of common variable immunodeficiency | Not relevant |
| Ozen et al | 2010 | Outcome of hypogammaglobulinemia in children: Immunoglobulin levels as predictors | Low N/cohort characteristics |
| Pac et al | 2019 | Clinical analysis of x-linked agammaglobulinaemia and common variable immunodeficiency in children - what should paediatricians know? | Low N/cohort characteristics |
| Papanastasiou et al | 2023 | Large-scale deep learning analysis to identify adult patients at risk for combined and common variable immunodeficiencies | Not relevant |
| Patel et al | 2019 | Interstitial lung disease in patients with common variable immunodeficiency disorders: Several different pathologies? | Low N/cohort characteristics |
| Patiroglu et al | 2012 | Autoimmune diseases detected in children with primary immunodeficiency diseases: Results from a reference centre at middle anatolia | Low N/cohort characteristics |
| Pehlivanoglu et al | 2019 | Gastrointestinal findings in 26 adults with common variable immunodeficiency: The fickle nature of the disease manifests in gastrointestinal biopsies | Low N/cohort characteristics |
| Periselneris et al | 2021 | Bronchiectasis severity correlates with outcome in patients with primary antibody deficiency | Not relevant |
| Popa et al | 2022 | Common variable immune deficiency: An outpatient experience | Study design/outcomes reported |
| Pott et al | 2022 | Primary antibody deficiency-associated arthritis shares features with spondyloarthritis and enteropathic arthritis | Study design/outcomes reported |
| Pruzanski et al | 1996 | Relationship of the dose of intravenous gammaglobulin to the prevention of infections in adults with common variable immunodeficiency | IRT/ treatment not relevant |
| Pulvirenti et al | 2014 | Idiopathic non cirrhotic portal hypertension and spleno-portal axis abnormalities in patients with severe primary antibody deficiencies | Not relevant |
| Reda et al | 2009 | Primary immunodeficiency diseases in egyptian children: A single-center study | Low N/cohort characteristics |
| Reisi et al | 2017 | Evaluation of pulmonary complications in patients with primary immunodeficiency disorders | Not relevant |
| Rezaei et al | 2008 | Increased serum levels of soluble cd30 in patients with common variable immunodeficiency and its clinical implications | Low N/cohort characteristics |
| Rubin et al | 2022 | Allergic-like disorders and asthma in patients with common variable immunodeficiency: A multi-center experience | Low N/cohort characteristics |
| Ruffner and Sullivan | 2018 | Complications associated with underweight primary immunodeficiency patients: Prevalence and associations within the usidnet registry | Not relevant |
| Ryser et al | 1988 | Primary immunodeficiencies in switzerland: First report of the national registry in adults and children | Study design/outcomes reported |
| Sanchez et al | 2023 | Characterization of infectious and non-infectious gastrointestinal disease in common variable immunodeficiency: Analysis of 114 patient cohort | Study design/outcomes reported |
| Scarpa et al | 2023 | Common and uncommon ct findings in cvid-related gl-ild: Correlations with clinical parameters, therapeutic decisions and potential implications in the differential diagnosis | Not relevant |
| Schutz et al | 2019 | Imaging of bronchial pathology in antibody deficiency: Data from the european chest ct group | Not relevant |
| Selenius et al | 2017 | Unexpectedly high prevalence of common variable immunodeficiency in finland | Not relevant |
| Shavit et al | 2021 | Combined immunodeficiency (cvid and cd4 lymphopenia) is associated with a high risk of malignancy among adults with primary immune deficiency | Low N/cohort characteristics |
| Sifers et al | 2020 | Vedolizumab therapy in common variable immune deficiency associated enteropathy: A case series | IRT/ treatment not relevant |
| Smith and Cunningham-Rundles | 2021 | Lymphoid malignancy in common variable immunodeficiency in a single-center cohort | Study design/outcomes reported |
| Smits et al | 2024 | Pulmonary computed tomography screening frequency in primary antibody deficiency | Not relevant |
| Somasundaram et al | 2023 | Clinical and immunological characterisation of patients with common variable immunodeficiency related immune thrombocytopenia | Low N/cohort characteristics |
| Sperlich et al | 2022 | Predictive factors for and complications of bronchiectasis in common variable immunodeficiency disorders | Study design/outcomes reported |
| Stuchly et al | 2017 | Common variable immunodeficiency patients with a phenotypic profile of immunosenescence present with thrombocytopenia | Not relevant |
| Tanir Basaranoglu et al | 2021 | Oxidative stress in common variable immunodeficiency | Study design/outcomes reported |
| Thickett et al | 2002 | Common variable immune deficiency: Respiratory manifestations, pulmonary function and high-resolution ct scan findings | Not relevant |
| Viallard et al | 2013 | Perturbations of the cd8<sup>+</sup> t-cell repertoire in cvid patients with complications | Low N/cohort characteristics |
| Vodjgani et al | 2007 | Analysis of class-switched memory b cells in patients with common variable immunodeficiency and its clinical implications | Low N/cohort characteristics |
| Wang et al | 2023 | High occurrence of autoimmune and lymphoproliferative manifestations in adults with common variable immunodeficiency in southerntaiwan | Low N/cohort characteristics |
| Wang et al | 2004 | Immunological and clinical features of pediatric patients with primary hypogammaglobulinemia in taiwan | Low N/cohort characteristics |
| Wang et al | 2011 | Distribution and clinical features of primary immunodeficiency diseases in chinese children (2004-2009) | Low N/cohort characteristics |
| Wiesik-Szewczyk et al | 2018 | The first polish cohort of adult patients with common variable immunodeficiency from 4 specialized centers: Do we provide standards of care? | Low N/cohort characteristics |
| Ylldlz et al | 2023 | Allergic diseases as a clinical phenotype marker in patients with common variable immunodeficiency | Study design/outcomes reported |

*CVID, common variable immune deficiency; IRT, immunoglobulin replacement therapy.*
