# Supplementary material for: A systematic literature review of CVID reveals pervasive detrimental noninfectious manifestations
Source: J Hum Immun. 2025 Oct 24;2(1):e20250157. doi: 10.70962/jhi.20250157 (PMC13177467; doi:10.70962/jhi.20250157)
Supplement: Table S4 — shows all manifestations with mortality HRs and P values. [file jhi_20250157_tables4.docx]

**Table S4. All manifestations with mortality hazard ratios and *P* values**

| Manifestation | HR (95% CI) | *P* value | Reference | Notes |
| --- | --- | --- | --- | --- |
| Any Non-Infectious Complication | | | | |
| Any Non-Infectious Complication | 10.96 (3.46-34.69) | 0.0001 | Resnick et al. (2012) | Multivariate |
| Splenomegaly | | | | |
| Splenomegaly | 2.11 (1.17-3.82) | 0.013 | Odnoletkova et al. (2018) | Univariate |
| Splenomegaly | 1.67 (0.82-3.39) | 0.155 | Odnoletkova et al. (2018) | Multivariate |
| Splenectomy | 1.69 (0.91-3.12) | 0.0957 | Resnick et al. (2012) | Multivariate |
| Lung Disease | | | | |
| GLILD | 2.14 (1.09-4.21) | 0.03 | Halliday et al. (2024) | Univariate |
| GLILD | 1.25 (0.56-2.80) | 0.58 | Halliday et al. (2024) | Multivariate |
| GLILD | 3.80 (1.47-9.85) | 0.006 | Odnoletkova et al. (2018) | Univariate |
| GLILD | 4.85 (1.63-14.39) | 0.005 | Odnoletkova et al. (2018) | Multivariate |
| Bronchiectasis | 0.84 (0.44-1.62) | 0.612 | Odnoletkova et al. (2018) | Univariate |
| Bronchiectasis | 0.83 (0.40-1.86) | 0.633 | Odnoletkova et al. (2018) | Multivariate |
| Bronchiectasis | 0.76 (0.39-1.48) | 0 .4235 | Resnick et al. (2012) | Multivariate |
| Chronic Lung Disease^*^ | 2.06 (1.34-3.16) | 0.0010 | Resnick et al. (2012) | Multivariate |
| GI Disease | | | | |
| Enteropathy | 1.39 (0.54-3.56) | 0.493 | Odnoletkova et al. (2018) | Univariate |
| Enteropathy | 0.97 (0.28-3.41) | 0.962 | Odnoletkova et al. (2018) | Multivariate |
| Gastrointestinal Disease | 2.78 (1.44-3.59) | 0.0004 | Resnick et al. (2012) | Multivariate |
| Malabsorption | 2.06 (1.11-3.81) | 0.0218 | Resnick et al. (2012) | Multivariate |
| Liver Disease | | | | |
| Liver-Related Abnormality^ꝉ^ | 3.5 (1.2-9.9) | 0.0195 | Azzu et al. (2019) | Assume Univariate |
| Cirrhosis With or Without Portal Hypertension | 4.2 (1.5-11.9) | 0.0005 | Azzu et al. (2019) | Assume Univariate |
| Liver Disease | 2.17 (1.12-4.21) | 0.02 | Halliday et al. (2024) | Univariate |
| Liver Disease | 2.24 (1.06-4.74) | 0.04 | Halliday et al. (2024) | Multivariate |
| Liver Disease/Hepatitis | 2.48 (1.51-4.09) | 0.0003 | Resnick et al. (2012) | Multivariate |
| Autoimmune Cytopenia | | | | |
| Autoimmune Cytopenia | 0.72 (0.22-2.35) | 0.591 | Odnoletkova et al. (2018) | Univariate |
| Autoimmune Cytopenia | 1.08 (0.33-3.57) | 0.897 | Odnoletkova et al. (2018) | Multivariate |
| Autoimmunity | | | | |
| Autoimmunity (Organ, Systemic) | 1.61 (0.79-3.27) | 0.187 | Odnoletkova et al. (2018) | Univariate |
| Autoimmunity (Organ, Systemic) | 1.52 (0.67-3.43) | 0.311 | Odnoletkova et al. (2018) | Multivariate |
| Autoimmunity | 1.36 (0.87-2.12) | 0.1735 | Resnick et al. (2012) | Multivariate |
| Rheumatologic Disease | | | | |
| Inflammatory Arthritis | 2.25 (1.05-4.80) | 0.04 | Halliday et al. (2024) | Univariate |
| Inflammatory Arthritis | 1.95 (0.88-4.34) | 0.10 | Halliday et al. (2024) | Multivariate |
| Granuloma | | | | |
| Granuloma | 1.27 (0.65-2.48) | 0.4939 | Resnick et al. (2012) | Multivariate |
| Malignancy | | | | |
| Lymphoma | 3.95 (1.81-8.66) | 0.001 | Odnoletkova et al. (2018) | Univariate |
| Lymphoma | 5.48 (2.36-12.71) | <0.0001 | Odnoletkova et al. (2018) | Multivariate |
| Lymphoma | 4.2 (2.8-44.4) | 0.001 | Pulvirenti et al. (2018) | Paper also had standardized mortality ratios for other cancers |
| Lymphoma | 2.44 (1.43-4.16) | 0.0010 | Resnick et al. (2012) | Multivariate |
| Solid Tumor | 3.19 (1.55-6.57) | 0.002 | Odnoletkova et al. (2018) | Univariate |
| Solid Tumor | 2.69 (1.10-6.57) | 0.030 | Odnoletkova et al. (2018) | Multivariate |
| Gastric Cancer | 4.8 (4.2-44.4) | <0.0001 | Pulvirenti et al. (2018) | Paper also had standardized mortality ratios for other cancers |
| Cancer | 1.51 (0.795-2.87) | 0.2084 | Resnick et al. (2012) | Multivariate |
| Age | | | | |
| Age at CVID Diagnosis | 1.06 (1.03-1.08) | <0.001 | Halliday et al. (2024) | Univariate |
| Age at CVID Diagnosis | 1.06 (1.03-1.09) | <0.001 | Halliday et al. (2024) | Multivariate |
| Age at Diagnosis | 1.04 (1.03-1.05) | <0.0001 | Odnoletkova et al. (2018) | Univariate |
| Age at Diagnosis | 1.05 (1.04-1.06) | <0.0001 | Odnoletkova et al. (2018) | Multivariate |
| Age at Symptom Onset | 1.04 (1.03-1.05) | <0.0001 | Odnoletkova et al. (2018) | Univariate |
| Age at Symptom Onset | 1.05 (1.04-1.06) | <0.0001 | Odnoletkova et al. (2018) | Multivariate |
| Diagnostic delay |  |  |  |  |
| Diagnostic delay adjusted for age at CVID diagnosis | 0.99 (0.97-1.01) | 0.221 | Odnoletkova et al. (2018) | Multivariate |
| Diagnostic delay adjusted for age of symptoms onset | 1.04 (1.02-1.06) | 0.0003 | Odnoletkova et al. (2018) | Multivariate |

^*^Structural and functional.

^ꝉ^At least one of: abnormal LFT profile, abnormal liver imaging, or abnormal liver histology.

*CI, confidence intervals; CVID, common variable immunodeficiency; GLILD, granulomatous lymphocytic interstitial lung disease; HR, hazard ratio.*

References

1. Cunningham-Rundles, C., J.-L. Casanova, and B. Boisson. 2023. Genetics and clinical phenotypes in common variable immunodeficiency. *Front.* *Genet.* 14:1272912. <https://doi.org/10.3389/fgene.2023.1272912>

1. Pescador Ruschel, M.A., and S. Vaqar. 2025. Common Variable Immunodeficiency. StatPearls, Treasure Island.
2. Ranjbarnejad, T., H. Abolhassani, R. Sherkat, M. Salehi, F. Ranjbarnejad,

N. Vatandoost, and M. Sharifi. 2025. Exploring monogenic, polygenic, and epigenetic models of common variable immunodeficiency. *Hum.* *Mutat.* 2025:1725906. <https://doi.org/10.1155/humu/1725906>

1. Ramirez, N.J., S. Posadas-Cantera, A. Caballero-Oteyza, N. Camacho- Ordonez, and B. Grimbacher. 2021. There is no gene for CVID - novel monogenetic causes for primary antibody deficiency. *Curr. Opin. Im-* *munol.* 72:176–185. <https://doi.org/10.1016/j.coi.2021.05.010>
2. Bonilla, F.A., I. Barlan, H. Chapel, B.T. Costa-Carvalho, C. Cunningham-Rundles, M.T. de la Morena, F.J. Espinosa-Rosales, L. Hammarstrom, S. Nonoyama, I. Quinti, et al. 2016. International consensus document (ICON): Common variable immunodeficiency disorders. *J. Allergy Clin.* *Immunol. Pract.* 4:38–59. <https://doi.org/10.1016/j.jaip.2015.07.025>

6. Seidel, M.G., G. Kindle, B. Gathmann, I. Quinti, M. Buckland, J. van Montfrans, R. Scheible, S. Rusch, L.M. Gasteiger, B. Grimbacher, et al. 2019. The European society for immunodeficiencies (ESID) registry working definitions for the clinical diagnosis of inborn errors of im- munity. *J. Allergy Clin. Immunol. Pract.* 7:1763–1770. <https://doi.org/10.1016/j.jaip.2019.02.004>

7. Bousfiha, A., A. Moundir, S.G. Tangye, C. Picard, L. Jeddane, W. Al-Herz,

C.C. Rundles, J.L. Franco, S.M. Holland, C. Klein, et al. 2022. The 2022 update of IUIS phenotypical classification for human inborn errors of immunity. *J. Clin. Immunol.* 42:1508–1520. [https://doi.org/10.1007/](https://doi.org/10.1007/s10875-022-01352-z) [s10875-022-01352-z](https://doi.org/10.1007/s10875-022-01352-z)

1. Oksenhendler, E., L. Gerard, C. Fieschi, M. Malphettes, G. Mouillot, R.

Jaussaud, J.F. Viallard, M. Gardembas, L. Galicier, N. Schleinitz, et al. 2008. Infections in 252 patients with common variable immunodefi- ciency. *Clin. Infect. Dis.* 46:1547–1554. <https://doi.org/10.1086/587669>

1. Chapel, H., M. Lucas, M. Lee, J. Bjorkander, D. Webster, B. Grimbacher, C. Fieschi, V. Thon, M.R. Abedi, and L. Hammarstrom. 2008. Common variable immunodeficiency disorders: Division into distinct clinical phenotypes. *Blood*. 112:277–286. [https://doi.org/10.1182/blood-2007-11](https://doi.org/10.1182/blood-2007-11-124545)[-124545](https://doi.org/10.1182/blood-2007-11-124545)
2. Ho, H.-E., and C. Cunningham-Rundles. 2020. Non-infectious compli- cations of common variable immunodeficiency: Updated clinical spec- trum, sequelae, and insights to pathogenesis. *Front. Immunol.* 11:149. <https://doi.org/10.3389/fimmu.2020.00149>
3. Lee, T.K., J.D. Gereige, and P.J. Maglione. 2021. State-of-the-art diag- nostic evaluation of common variable immunodeficiency. *Ann. Allergy* *Asthma Immunol.* 127:19–27. <https://doi.org/10.1016/j.anai.2021.03.005>
4. Farmer, J.R., M.-S. Ong, S. Barmettler, L.M. Yonker, R. Fuleihan, K.E. Sullivan, C. Cunningham-Rundles, USIDNET Consortium, and J.E. Walter. 2017. Common variable immunodeficiency non-infectious dis- ease endotypes redefined using unbiased network clustering in large electronic datasets. *Front. Immunol.* 8:1740. [https://doi.org/10.3389/](https://doi.org/10.3389/fimmu.2017.01740)[fimmu.2017.01740](https://doi.org/10.3389/fimmu.2017.01740)
5. Thalhammer, J., G. Kindle, A. Nieters, S. Rusch, M.R.J. Seppa¨nen, A.

Fischer, B. Grimbacher, D. Edgar, M. Buckland, N. Mahlaoui, et al. 2021. Initial presenting manifestations in 16,486 patients with inborn errors of immunity include infections and noninfectious manifestations. *J. Allergy Clin. Immunol.* 148:1332–1341.e5. [https://doi.org/10.1016/j.jaci](https://doi.org/10.1016/j.jaci.2021.04.015)[.2021.04.015](https://doi.org/10.1016/j.jaci.2021.04.015)

1. Abolhassani, H., C.K. Lim, A. Aghamohammadi, and L. Hammarstro¨m. 2020. Histocompatibility complex status and mendelian randomization analysis in unsolved antibody deficiency. *Front. Immunol.* 11:14. [https://](https://doi.org/10.3389/fimmu.2020.00014) [doi.org/10.3389/fimmu.2020.00014](https://doi.org/10.3389/fimmu.2020.00014)
2. Abolhassani, H., L. Hammarstro¨m, and C. Cunningham-Rundles. 2020. Current genetic landscape in common variable immune deficiency. *Blood*. 135:656–667. <https://doi.org/10.1182/blood.2019000929>
3. Cunningham-Rundles, C. 2010. How I treat common variable immune deficiency. *Blood*. 116:7–15. [https://doi.org/10.1182/blood-2010-01](https://doi.org/10.1182/blood-2010-01-254417)[-254417](https://doi.org/10.1182/blood-2010-01-254417)
4. Abolhassani, H., B.T. Sagvand, T. Shokuhfar, B. Mirminachi, N. Rezaei, and A. Aghamohammadi. 2013. A review on guidelines for management and treatment of common variable immunodeficiency. *Expert Rev. Clin.* *Immunol.* 9:561–575. <https://doi.org/10.1586/eci.13.30>
5. Fevang, B. 2023. Treatment of inflammatory complications in common variable immunodeficiency (CVID): Current concepts and future per- spectives. *Expert Rev. Clin. Immunol.* 19:627–638. [https://doi.org/10](https://doi.org/10.1080/1744666X.2023.2198208)[.1080/1744666X.2023.2198208](https://doi.org/10.1080/1744666X.2023.2198208)
6. Brent, J., D. Guzman, C. Bangs, B. Grimbacher, C. Fayolle, A. Huissoon, C. Bethune, M. Thomas, S. Patel, S. Jolles, et al. 2016. Clinical and labora- tory correlates of lung disease and cancer in adults with idiopathic hypogammaglobulinaemia. *Clin. Exp. Immunol.* 184:73–82. [https://doi](https://doi.org/10.1111/cei.12748)[.org/10.1111/cei.12748](https://doi.org/10.1111/cei.12748)
7. Feuille, E.J., N. Anooshiravani, K.E. Sullivan, R.L. Fuleihan, and C. Cunningham-Rundles. 2018. Autoimmune cytopenias and associated conditions in CVID: A report from the USIDNET registry. *J. Clin. Im-* *munol.* 38:28–34. <https://doi.org/10.1007/s10875-017-0456-9>
8. Franzblau, L.E., R.L. Fuleihan, C. Cunningham-Rundles, and C.A. Wy- socki. 2023. CVID-associated intestinal disorders in the USIDNET reg- istry: An analysis of disease manifestations, functional status, comorbidities, and treatment. *J. Clin. Immunol.* 44:32. [https://doi.org/10](https://doi.org/10.1007/s10875-023-01604-6)[.1007/s10875-023-01604-6](https://doi.org/10.1007/s10875-023-01604-6)
9. Gathmann, B., N. Mahlaoui, CEREDIH, L. Gerard, E. Oksenhendler, K. Warnatz, I. Schulze, G. Kindle, T.W. Kuijpers, Dutch WID, et al. 2014. Clinical picture and treatment of 2212 patients with common variable immunodeficiency. *J. Allergy Clin. Immunol.* 134:116–126. [https://doi.org/](https://doi.org/10.1016/j.jaci.2013.12.1077) [10.1016/j.jaci.2013.12.1077](https://doi.org/10.1016/j.jaci.2013.12.1077)
10. Odnoletkova, I., G. Kindle, I. Quinti, B. Grimbacher, V. Knerr, B. Gathmann, S. Ehl, N. Mahlaoui, P. Van Wilder, K. Bogaerts, et al. 2018. The burden of common variable immunodeficiency disorders: A retro- spective analysis of the European society for immunodeficiency (ESID) registry data. *Orphanet J. Rare Dis.* 13:201. [https://doi.org/10.1186/s13023](https://doi.org/10.1186/s13023-018-0941-0)[-018-0941-0](https://doi.org/10.1186/s13023-018-0941-0)
11. Azizi, G., Y. Bagheri, M. Tavakol, F. Askarimoghaddam, K. Porrostami, H. Rafiemanesh, R. Yazdani, F. Kiaee, S. Habibi, K. Abouhamzeh, et al. 2018. The Clinical and immunological features of patients with primary antibody deficiencies. *Endocr. Metab. Immune Disord. Drug Targets*. 18: 537–545. <https://doi.org/10.2174/1871530318666180413110216>
12. Baloh, C., A. Reddy, M. Henson, K. Prince, R. Buckley, and P. Lugar. 2019. 30-year review of pediatric- and adult-onset CVID: Clinical cor- relates and prognostic indicators. *J. Clin. Immunol.* 39:678–687. [https://](https://doi.org/10.1007/s10875-019-00674-9) [doi.org/10.1007/s10875-019-00674-9](https://doi.org/10.1007/s10875-019-00674-9)
13. Aghamohammadi, A., H. Abolhassani, A. Latif, F. Tabassomi, T. Sho- kuhfar, B. Torabi Sagvand, S. Shahinpour, B. Mirminachi, N. Parvaneh, M. Movahedi, et al. 2014. Long-term evaluation of a historical cohort of Iranian common variable immunodeficiency patients. *Expert Rev. Clin.* *Immunol.* 10:1405–1417. <https://doi.org/10.1586/1744666X.2014.958469>
14. Boursiquot, J.-N., L. Ge´rard, M. Malphettes, C. Fieschi, L. Galicier, D. Boutboul, R. Borie, J.F. Viallard, P. Soulas-Sprauel, A. Berezne, et al. 2013. Granulomatous disease in CVID: Retrospective analysis of clinical characteristics and treatment efficacy in a cohort of 59 patients. *J. Clin.* *Immunol.* 33:84–95. <https://doi.org/10.1007/s10875-012-9778-9>
15. Cabañero-Navalon, M.D., V. Garcia-Bustos, M. Nuñez-Beltran, P. C´ıscar Ferna´ndez, L. Mateu, X. Solanich, J.L. Carrillo-Linares, A. Robles-Mar- huenda, F. Puchades-Gimeno, A. Pelaez Ballesta, et al. 2022. Current clinical spectrum of common variable immunodeficiency in Spain: The multicentric nationwide GTEM-SEMI-CVID registry. *Front. Immunol.* 13: 1033666. <https://doi.org/10.3389/fimmu.2022.1033666>
16. Cinetto, F., R. Scarpa, M. Carrabba, D. Firinu, V. Lougaris, H. Buso, G. Garzi, S. Gianese, V. Soccodato, A. Punziano, et al. 2021. Granulomatous lymphocytic interstitial lung disease (GLILD) in common variable im- munodeficiency (CVID): A multicenter retrospective study of patients from Italian PID referral centers. *Front. Immunol.* 12:627423. [https://doi](https://doi.org/10.3389/fimmu.2021.627423)[.org/10.3389/fimmu.2021.627423](https://doi.org/10.3389/fimmu.2021.627423)
17. Cunningham-Rundles, C. 1989. Clinical and immunologic analyses of 103 patients with common variable immunodeficiency. *J. Clin. Immunol.* 9:22–33. <https://doi.org/10.1007/BF00917124>
18. Cunningham-Rundles, C., and C. Bodian. 1999. Common variable im- munodeficiency: Clinical and immunological features of 248 patients. *Clin. Immunol.* 92:34–48. <https://doi.org/10.1006/clim.1999.4725>
19. Esmaeilzadeh, H., A. Jokar-Derisi, A.H. Hassani, R. Yazdani, S. Delavari, H. Abolhassani, N. Mortazavi, and A. Askarisarvestani. 2023. Assess- ment of the first presentations of common variable immunodeficiency in a large cohort of patients. *BMC Immunol.* 24:9. [https://doi.org/10](https://doi.org/10.1186/s12865-023-00545-4)[.1186/s12865-023-00545-4](https://doi.org/10.1186/s12865-023-00545-4)
20. Filion, C.A., S. Taylor-Black, P.J. Maglione, L. Radigan, and C. Cun- ningham-Rundles. 2019. Differentiation of common variable immuno- deficiency from IgG deficiency. *J. Allergy Clin. Immunol. Pract.* 7: 1277–1284. <https://doi.org/10.1016/j.jaip.2018.12.004>
21. Ghorbani, M., S. Fekrvand, S. Shahkarami, R. Yazdani, M. Sohani, M. Shaghaghi, G. Hassanpour, J. Mohammadi, B. Negahdari, H. Abolhas- sani, and A. Aghamohammadi. 2019. The evaluation of neutropenia in common variable immune deficiency patients. *Expert Rev. Clin. Im**munol.* 15:1225–1233. <https://doi.org/10.1080/1744666X.2020.1677154>
22. Gouilleux-Gruart, V., H. Chapel, S. Chevret, M. Lucas, M. Malphettes, C. Fieschi, S. Patel, D. Boutboul, M.-N. Marson, L. Gerard, et al. 2013. Ef- ficiency of immunoglobulin G replacement therapy in common variable immunodeficiency: Correlations with clinical phenotype and poly- morphism of the neonatal Fc receptor. *Clin. Exp. Immunol.* 171:186–194. <https://doi.org/10.1111/cei.12002>
23. Halliday, N., N. Eden, H. Somers, N. Burke, H. Silva, C.G. Brito, A. Hall, A. Quaglia, S.O. Burns, D.M. Lowe, and D. Thorburn. 2024. Common variable immunodeficiency disorder-related liver disease is common and results in portal hypertension and an increased risk of death. *Hepatol. Commun.* 8:e0322. [https://doi.org/10.1097/HC9](https://doi.org/10.1097/HC9.0000000000000322)[.0000000000000322](https://doi.org/10.1097/HC9.0000000000000322)
24. Hanitsch, L.G., S. Steiner, M. Schumann, K. Wittke, C. Kedor, C. Scheibenbogen, and A. Fischer. 2023. Portal hypertension in common variable immunodeficiency disorders - a single center analysis on clinical and immunological parameter in 196 patients. *Front. Immunol.* 14:1268207. <https://doi.org/10.3389/fimmu.2023.1268207>
25. Jørgensen, S.F., H.M. Reims, D. Frydenlund, K. Holm, V. Paulsen, A.E. Michelsen, K.K. Jørgensen, L.T. Osnes, J. Bratlie, T.J. Eide, et al. 2016. A cross-sectional study of the prevalence of gastrointestinal symptoms and pathology in patients with common variable immunodeficiency. *Am. J. Gastroenterol.* 111:1467–1475. <https://doi.org/10.1038/ajg.2016.329>
26. Mohammadinejad, P., S. Pourhamdi, H. Abolhassani, B. Mirminachi, A. Havaei, S.N. Masoom, B. Sadeghi, A. Ghajar, M. Afarideh, N. Parvaneh, et al. 2015. Primary antibody deficiency in a tertiary referral hospital: A 30-year experiment. *J. Investig. Allergol. Clin. Immunol.* 25:416–425.
27. Pashangzadeh, S., S. Delavari, T. Moeini Shad, F. Salami, S.E. Rasouli, R. Yazdani, S.A. Mahdaviani, M. Nabavi, S. Aleyasin, H. Ahanchian, et al. 2024. Noninfectious complications in B-lymphopenic common variable immunodeficiency. *J. Investig. Allergol. Clin. Immunol.* 34:233–245. <https://doi.org/10.18176/jiaci.0902>
28. Pulvirenti, F., A. Pecoraro, F. Cinetto, C. Milito, M. Valente, E. Santan- geli, L. Crescenzi, F. Rizzo, S. Tabolli, G. Spadaro, et al. 2018. Gastric cancer is the leading cause of death in Italian adult patients with com- mon variable immunodeficiency. *Front. Immunol.* 9:2546. [https://doi](https://doi.org/10.3389/fimmu.2018.02546)[.org/10.3389/fimmu.2018.02546](https://doi.org/10.3389/fimmu.2018.02546)
29. Quinti, I., A. Soresina, A. Guerra, R. Rondelli, G. Spadaro, C. Agostini, C. Milito, A.C. Trombetta, M. Visentini, H. Martini, et al. 2011. Effective- ness of immunoglobulin replacement therapy on clinical outcome in patients with primary antibody deficiencies: Results from a multicenter prospective cohort study. *J. Clin. Immunol.* 31:315–322. [https://doi.org/10](https://doi.org/10.1007/s10875-011-9511-0)[.1007/s10875-011-9511-0](https://doi.org/10.1007/s10875-011-9511-0)
30. Quinti, I., A. Soresina, G. Spadaro, S. Martino, S. Donnanno, C. Agostini, P. Claudio, D. Franco, A. Maria Pesce, F. Borghese, et al. 2007. Long-term follow-up and outcome of a large cohort of patients with common variable immunodeficiency. *J. Clin. Immunol.* 27:308–316. [https://doi](https://doi.org/10.1007/s10875-007-9075-1)[.org/10.1007/s10875-007-9075-1](https://doi.org/10.1007/s10875-007-9075-1)
31. Resnick, E.S., E.L. Moshier, J.H. Godbold, and C. Cunningham-Rundles. 2012. Morbidity and mortality in common variable immune deficiency over 4 decades. *Blood*. 119:1650–1657. [https://doi.org/10.1182/blood-2011](https://doi.org/10.1182/blood-2011-09-377945)[-09-377945](https://doi.org/10.1182/blood-2011-09-377945)
32. Sanchez, L.A., S.M. Maggadottir, M.S. Pantell, P. Lugar, C.C. Rundles, K.E. Sullivan. and USIDNET Consortium. 2017. Two sides of the same coin: Pediatric-onset and adult-onset common variable immune defi- ciency. *J. Clin. Immunol.* 37:592–602. [https://doi.org/10.1007/s10875-017](https://doi.org/10.1007/s10875-017-0415-5)[-0415-5](https://doi.org/10.1007/s10875-017-0415-5)
33. Slade, C.A., J.J. Bosco, T. Binh Giang, E. Kruse, R.G. Stirling, P.U. Ca meron, F. Hore-Lacy, M.F. Sutherland, S.L. Barnes, S. Holdsworth, et al. 2018. Delayed diagnosis and complications of predominantly antibody deficiencies in a cohort of australian adults. *Front. Immunol.* 9:694. <https://doi.org/10.3389/fimmu.2018.00694>
34. Valizadeh, A., R. Yazdani, G. Azizi, H. Abolhassani, and A. Aghamohammadi. 2017. A comparison of clinical and immunologic phenotypes in familial and sporadic forms of common variable immunodeficiency. *Scand. J. Immunol.* 86:239–247. <https://doi.org/10.1111/sji.12593>
35. von Spee-Mayer, C., V. Koemm, C. Wehr, S. Goldacker, G. Kindle, A. Bulashevska, M. Proietti, B. Grimbacher, S. Ehl, and K. Warnatz. 2019. Evaluating laboratory criteria for combined immunodeficiency in adult patients diagnosed with common variable immunodeficiency. *Clin.* *Immunol.* 203:59–62. <https://doi.org/10.1016/j.clim.2019.04.001>
36. Wehr, C., T. Kivioja, C. Schmitt, B. Ferry, T. Witte, E. Eren, M. Vlkova, M. Hernandez, D. Detkova, P.R. Bos, et al. 2008. The EUROclass trial: Defining subgroups in common variable immunodeficiency. *Blood*. 111: 77–85. <https://doi.org/10.1182/blood-2007-06-091744>
37. Westh, L., T.H. Mogensen, L.S. Dalgaard, J.M. Bernth Jensen, T. Kat- zenstein, A.-B.E. Hansen, O.D. Larsen, S. Terpling, T.L. Nielsen, and C.S. Larsen. 2017. Identification and characterization of a Nationwide Dan- ish adult common variable immunodeficiency cohort. *Scand. J. Immunol.* 85:450–461. <https://doi.org/10.1111/sji.12551>
38. Zarezadeh Mehrabadi, A., N. Aghamohamadi, H. Abolhassani, A. Aghamohammadi, N. Rezaei, and R. Yazdani. 2022. Comprehensive assessment of skin disorders in patients with common variable immu- nodeficiency (CVID). *J. Clin. Immunol.* 42:653–664. [https://doi.org/10](https://doi.org/10.1007/s10875-022-01211-x)[.1007/s10875-022-01211-x](https://doi.org/10.1007/s10875-022-01211-x)
39. Zie˛tkiewicz, M., E. Wie˛sik-Szewczyk, A. Matyja-Bednarczyk, K. Na- piórkowska-Baran, Z. Zdrojewski, and K. Jahnz-Róz˙yk. 2020. Shorter diagnostic delay in polish adult patients with common variable immu- nodeficiency and symptom onset after 1999. *Front. Immunol.* 11:982. <https://doi.org/10.3389/fimmu.2020.00982>
40. Ameratunga, R., A. Jordan, A. Cavadino, S. Ameratunga, T. Hills, R. Steele, M. Hurst, B. McGettigan, I. Chua, M. Brewerton, et al. 2021. Bronchiectasis is associated with delayed diagnosis and adverse out- comes in the New Zealand common variable immunodeficiency dis- orders cohort study. *Clin. Exp. Immunol.* 204:352–360. [https://doi.org/10](https://doi.org/10.1111/cei.13595)[.1111/cei.13595](https://doi.org/10.1111/cei.13595)
41. Dahl, C., I. Petersen, F.V. Ilkjær, L. Westh, T.L. Katzenstein, A.-B.E. Hansen, T.L. Nielsen, C.S. Larsen, I.S. Johansen, and L.D. Rasmussen. 2023. Missed opportunities to diagnose common variable immunode- ficiency: A population-based case-control study identifying indicator diseases for common variable immunodeficiency. *J. Clin. Immunol.* 43: 2104–2114. <https://doi.org/10.1007/s10875-023-01590-9>
42. 2022. Autoimmunity, t-large granluar lymphocytes (t-lgl) and switched memory b cells (smb) in common variable immunodeficiency (CVID). European Society for Immunodeficiencies Biennial Meeting 2022, Gothenburgh.
43. 2022. Autoimmune manifestations in a large multi-centre cohort of patients with common variable immunodeficiency in India. European Society for Immunodeficiencies Biennial Meeting 2022, Gothenburgh.
44. Aghamohammadi, A., N. Pouladi, N. Parvaneh, M. Yeganeh, M. Mova- hedi, M. Gharagolou, Z. Pourpak, N. Rezaei, A. Salavati, S. Abdollah- zade, and M. Moin. 2007. Mortality and morbidity in common variable immunodeficiency. *J. Trop. Pediatr.* 53:32–38. [https://doi.org/10.1093/](https://doi.org/10.1093/tropej/fml077) [tropej/fml077](https://doi.org/10.1093/tropej/fml077)
45. Azzu, V., M. Fonseca, A. Duckworth, L. Kennard, N. Moini, M. Qurashi, R. Brais, S. Davies, A. Manson, E. Staples, et al. 2019. Liver disease is common in patients with common variable immunodeficiency and predicts mortality in the presence of cirrhosis or portal hypertension. *J. Allergy Clin. Immunol. Pract.* 7:2484–2486.e3. [https://doi.org/10.1016/j](https://doi.org/10.1016/j.jaip.2019.04.016)[.jaip.2019.04.016](https://doi.org/10.1016/j.jaip.2019.04.016)
46. Aygun, A., E. Topyıldız, M. Geyik, N.E. Karaca, A. Durmaz, G. Aksu, A. Aykut, and N. Kutukculer. 2024. Current genetic defects in common variable immunodeficiency patients on the geography between Europe and Asia: A single-center experience. *Immunol. Res.* 72:225–233. [https://](https://doi.org/10.1007/s12026-023-09426-9) [doi.org/10.1007/s12026-023-09426-9](https://doi.org/10.1007/s12026-023-09426-9)
47. Bates, C.A., M.C. Ellison, D.A. Lynch, C.D. Cool, K.K. Brown, and J.M. Routes. 2004. Granulomatous-lymphocytic lung disease shortens sur- vival in common variable immunodeficiency. *J. Allergy Clin. Immunol.* 114:415–421. <https://doi.org/10.1016/j.jaci.2004.05.057>
48. Carrabba, M., M. Salvi, L.A. Baselli, S. Serafino, M. Zarantonello, E. Trombetta, M.C. Pietrogrande, G. Fabio, and R.M. Dellepiane. 2023. Long-term follow-up in common variable immunodeficiency: The pediatric-onset and adult-onset landscape. *Front. Pediatr.* 11:1125994. <https://doi.org/10.3389/fped.2023.1125994>
49. Crescenzi, L., A. Pecoraro, A. Fiorentino, R. Poto, G. Varricchi, A. Rispo, F. Morisco, and G. Spadaro. 2019. Liver stiffness assessment by transient elastography suggests high prevalence of liver involvement in common variable immunodeficiency. *Dig. Liver Dis.* 51:1599–1603. [https://doi](https://doi.org/10.1016/j.dld.2019.05.016)[.org/10.1016/j.dld.2019.05.016](https://doi.org/10.1016/j.dld.2019.05.016)
50. Daza-Cajigal, V., M. Segura-Guerrero, M. López-Cueto, A´. Robles-Marhuenda, C. Camara, T. Gerra-Gala´n, R. Gómez-de-la-Torre, C.L. Avendaño-Monje, S. Sanchez-Ramon, M.J. Bosque-Lopez, et al. 2023. Clinical manifestations and approach to the management of patients with common variable immunodeficiency and liver disease. *Front. Im* *munol.* 14:1197361. <https://doi.org/10.3389/fimmu.2023.1197361>
51. Fortier, J.C., E. Haltigan, V. Cavero-Chavez, D. Gomez-Manjarres, J.D. Squire, W.H. Reeves, and L. Cuervo-Pardo. 2022. Clinical and pheno- typic characterization of common variable immunodeficiency diag- nosed in younger and older adults. *J. Clin. Immunol.* 42:1270–1279. <https://doi.org/10.1007/s10875-022-01290-w>
52. Friedmann, D., B. Keller, I. Harder, J. Schupp, Y. Tanriver, S. Unger, and K. Warnatz. 2017. Preferential reduction of circulating innate lymphoid cells type 2 in patients with common variable immunodeficiency with secondary complications is part of a broader immune dysregulation. *J. Clin. Immunol.* 37:759–769. <https://doi.org/10.1007/s10875-017-0444-0>
53. Graziano, V., A. Pecoraro, I. Mormile, G. Quaremba, A. Genovese, C. Buccelli, M. Paternoster, and G. Spadaro. 2017. Delay in diagnosis affects the clinical outcome in a cohort of cvid patients with marked reduction of iga serum levels. *Clin. Immunol.* 180:1–4. [https://doi.org/10.1016/j](https://doi.org/10.1016/j.clim.2017.03.011)[.clim.2017.03.011](https://doi.org/10.1016/j.clim.2017.03.011)
54. Kainulainen, L., J. Nikoskelainen, and O. Ruuskanen. 2001. Diagnostic findings in 95 Finnish patients with common variable immunodeficiency. *J. Clin. Immunol.* 21:145–149. https://doi.org/10.1023/a: 1011012023616
55. Lucas, M., M. Lee, J. Lortan, E. Lopez-Granados, S. Misbah, and H. Chapel. 2010. Infection outcomes in patients with common variable immunodeficiency disorders: Relationship to immunoglobulin therapy over 22 years. *J. Allergy Clin. Immunol.* 125:1354–1360.e4. [https://doi.org/](https://doi.org/10.1016/j.jaci.2010.02.040) [10.1016/j.jaci.2010.02.040](https://doi.org/10.1016/j.jaci.2010.02.040)
56. Maarschalk-Ellerbroek, L.J., A.I.M. Hoepelman, J.M. van Montfrans, and P.M. Ellerbroek. 2012. The spectrum of disease manifestations in patients with common variable immunodeficiency disorders and partial antibody deficiency in a university hospital. *J. Clin. Immunol.* 32: 907–921. <https://doi.org/10.1007/s10875-012-9671-6>

70. Macpherson, M.E., T. Skarpengland, J.R. Hov, T. Ranheim, B. Vestad, T.B. Dahl, M.S.A. Fraz, A.E. Michelsen, K.B. Holven, B. Fevang, et al. 2023. Increased plasma levels of triglyceride-enriched lipoproteins as- sociate with systemic inflammation, lipopolysaccharides, and gut dys- biosis in common variable immunodeficiency. *J. Clin. Immunol.* 43: 1229–1240. <https://doi.org/10.1007/s10875-023-01475-x>

1. Maglione, P.J., J.R. Overbey, L. Radigan, E. Bagiella, and C. Cunningham- Rundles. 2014. Pulmonary radiologic findings in common variable im- munodeficiency: Clinical and immunological correlations. *Ann. Allergy Asthma Immunol.* 113:452–459. [https://doi.org/10.1016/j.anai.2014.04](https://doi.org/10.1016/j.anai.2014.04.024)[.024](https://doi.org/10.1016/j.anai.2014.04.024)
2. Mannina, A., J.H. Chung, J.J. Swigris, J.J. Solomon, T.J. Huie, Z.X. Yunt, T.Q. Truong, K.K. Brown, R.D. Achcar, A.L. Olson, et al. 2016. Clinical predictors of a diagnosis of common variable immunodeficiency-related granulomatous-lymphocytic interstitial lung disease. *Ann. Am. Thorac.* *Soc.* 13:1042–1049. <https://doi.org/10.1513/AnnalsATS.201511-728OC>
3. Markocsy, A., A. Bobcakova, O. Petrovicova, L. Kapustova, E. Ma- licherova Jurkova, M. Schniederova, J. Petriskova, M. Cibulka, M. Hy- blova, and M. Jesenak. 2024. Association between cytometric biomarkers, clinical phenotype, and complications of common variable immunodeficiency. *Cureus*. 16:e52941. [https://doi.org/10.7759/cureus](https://doi.org/10.7759/cureus.52941)[.52941](https://doi.org/10.7759/cureus.52941)
4. Mohammadinejad, P., A. Aghamohammadi, H. Abolhassani, M.S. Sa- daghiani, S. Abdollahzade, B. Sadeghi, H. Soheili, M. Tavassoli, S.M. Fathi, M. Tavakol, et al. 2012. Pediatric patients with common variable immunodeficiency: Long-term follow-up. *J. Investig. Allergol. Clin. Im**munol.* 22:208–214.
5. Mormile, I., A. Punziano, C.A. Riolo, F. Granata, M. Williams, A. de Paulis, G. Spadaro, and F.W. Rossi. 2021. Common variable immuno- deficiency and autoimmune diseases: A retrospective study of 95 adult patients in a single tertiary care center. *Front. Immunol.* 12:652487. <https://doi.org/10.3389/fimmu.2021.652487>
6. Pedini, V., J.U. Verga, I. Terrenato, D. Menghini, C. Mezzanotte, and M.G. Danieli. 2020. Incidence of malignancy in patients with common variable immunodeficiency according to therapeutic delay: An Italian retrospective, monocentric cohort study. *Allergy Asthma. Clin. Immunol.* 16:54. <https://doi.org/10.1186/s13223-020-00451-z>
7. Viallard, J.-F., M. Parrens, P. Blanco, J.-F. Moreau, E. Oksenhendler, and C. Fieschi. 2024. Influence of splenomegaly and Splenectomy on the immune cell profile of patients with common variable immunodefi- ciency disease. *J. Clin. Immunol.* 44:46. [https://doi.org/10.1007/s10875](https://doi.org/10.1007/s10875-023-01648-8)[-023-01648-8](https://doi.org/10.1007/s10875-023-01648-8)
8. Wang, J., and C. Cunningham-Rundles. 2005. Treatment and outcome of autoimmune hematologic disease in common variable immunodefi- ciency (CVID). *J. Autoimmun.* 25:57–62. [https://doi.org/10.1016/j.jaut](https://doi.org/10.1016/j.jaut.2005.04.006)[.2005.04.006](https://doi.org/10.1016/j.jaut.2005.04.006)
9. Khan, R., M. Habbal, M.A. Scaffidi, A.A. Bukhari, A. Rumman, S. Al Ghamdi, S.D. Betschel, and S.C. Grover. 2020. Gastrointestinal disease in patients with common variable immunodeficiency: A retrospective observational study. *J. Can. Assoc. Gastroenterol.* 3:162–168. [https://doi](https://doi.org/10.1093/jcag/gwz004)[.org/10.1093/jcag/gwz004](https://doi.org/10.1093/jcag/gwz004)
10. Arshi, S., M. Nabavi, M.H. Bemanian, R. Shakeri, B. Taghvaei, B. Gha- lebaghi, D. Babaie, A. Bahrami, M. Fallahpour, H. Esmaeilzadeh, et al. 2016. Phenotyping and follow up of forty-seven Iranian patients with common variable immunodeficiency. *Allergol. Immunopathol.* 44: 226–231. <https://doi.org/10.1016/j.aller.2015.04.005>
11. Bazregari, S., G. Azizi, M. Tavakol, M.H. Asgardoon, F. Kiaee, N. Tavakolinia, A. Valizadeh, H. Abolhassani, and A. Aghamohammadi. 2017. Evaluation of infectious and non-infectious complications in patients with primary immunodeficiency. *Cent. Eur. J. Immunol.* 42:336–341. <https://doi.org/10.5114/ceji.2017.72825>
12. Bezrodnik, L., M.I. Gaillard, and D. Carelli. 2011. Clinical and immuno- logical assessment of 94 patients with primary humoral immunodefi- ciency: Common variable immunodeficiency, selective iga deficiency and polysaccharide antibody deficiency syndrome. *J. Pediatr. Infect. Dis.* 6:159–166. <https://doi.org/10.3233/JPI-2011-0320>
13. Desjardins, M., M. Be´land, M. Dembele, D. Lejtenyi, J.P. Drolet, M. Lemire, C. Tsoukas, M. Ben-Shoshan, F.J.D. Noya, R. Alizadehfar, et al. 2018. Modulation of the Interleukin-21 pathway with Interleukin-4 distinguishes common variable immunodeficiency patients with more non-infectious clinical complications. *J. Clin. Immunol.* 38:45–55. <https://doi.org/10.1007/s10875-017-0452-0>
14. Fraz, M.S.A., N. Moe, M.E. Revheim, M.L. Stavrinou, M.T. Durheim, I. Nordøy, M.E. Macpherson, P. Aukrust, S.F. Jørgensen, T.M. Aaløkken, and B. Fevang. 2020. Granulomatous-lymphocytic interstitial lung disease in common variable immunodeficiency-features of CT and (18)F-FDG positron emission tomography/CT in clinically progressive disease. *Front. Immunol.* 11:617985. [https://doi.org/10.3389/fimmu.2020](https://doi.org/10.3389/fimmu.2020.617985)[.617985](https://doi.org/10.3389/fimmu.2020.617985)
15. Gobert, D., J.B. Bussel, C. Cunningham-Rundles, L. Galicier, A. De- chartres, A. Berezne, B. Bonnotte, T. DeRevel, C. Auzary, R. Jaussaud, et al. 2011. Efficacy and safety of rituximab in common variable immunodeficiency-associated immune cytopenias: A retrospective multicentre study on 33 patients. *Br. J. Haematol.* 155:498–508. [https://](https://doi.org/10.1111/j.1365-2141.2011.08880.x) [doi.org/10.1111/j.1365-2141.2011.08880.x](https://doi.org/10.1111/j.1365-2141.2011.08880.x)
16. Lopes, J.P., H.-E. Ho, and C. Cunningham-Rundles. 2021. Interstitial lung disease in common variable immunodeficiency. *Front. Immunol.* 12: 605945. <https://doi.org/10.3389/fimmu.2021.605945>
17. Maarschalk-Ellerbroek, L.J., P.A. de Jong, J.M. van Montfrans, J.W.J. Lammers, A.C. Bloem, A.I.M. Hoepelman, and P.M. Ellerbroek. 2014. CT screening for pulmonary pathology in common variable immunodefi- ciency disorders and the correlation with clinical and immunological parameters. *J. Clin. Immunol.* 34:642–654. [https://doi.org/10.1007/](https://doi.org/10.1007/s10875-014-0068-6)[s10875-014-0068-6](https://doi.org/10.1007/s10875-014-0068-6)
18. Mus¸abak, U.H., F. Demirel, S. Yes¸illik, A. Baysan, A. Selçuk, O. Kartal, M. Güleç, Ç. O¨ ktenli, and O. S¸ener. 2017. Adults with common variable immunodeficiency: A single-center experience. *Turk J. Med. Sci.* 47:1–12. <https://doi.org/10.3906/sag-1503-22>
19. Pia˛tosa, B., M. Pac, K. Siewiera, B. Pietrucha, M. Klaudel-Dreszler, E. Heropolitan´ska-Pliszka, B. Wolska-Kus´nierz, H. Dmenska, H. Gregorek, I. Sokolnicka, et al. 2013. Common variable immune deficiency in children--clinical characteristics varies depending on defect in pe- ripheral B cell maturation. *J. Clin. Immunol.* 33:731–741. [https://doi.org/](https://doi.org/10.1007/s10875-013-9875-4) [10.1007/s10875-013-9875-4](https://doi.org/10.1007/s10875-013-9875-4)
20. Ramırez-Vargas, N., S.E. Arablin-Oropeza, D. Mojica-Mart´ınez, M.A. Yamazaki-Nakashimada, M. de la Luz Garcia-Cruz, L.M. Teran-Juarez, R.M. Cortes-Grimaldo, C. Torres-Lozano, I. Madrigal-Beas, M. Ortega-Cisneros, et al. 2014. Clinical and immunological features of common variable immunodeficiency in Mexican patients. *Allergol. Immunopathol* *(Madr).* 42:235–240. <https://doi.org/10.1016/j.aller.2013.01.007>
21. Szczawin´ska-Popłonyk, A., K. Ta Polska-Józ´wiak, E. Schwartzmann, and N. Popłonyk. 2022. Immune Dysregulation in pediatric common variable immunodeficiency: Implications for the diagnostic approach. *Front. Pediatr.* 10:855200. <https://doi.org/10.3389/fped.2022.855200>
22. Turpin, D., A. Furudoi, M. Parrens, P. Blanco, J.F. Viallard, and D. Duluc. 2018. Increase of follicular helper T cells skewed toward a Th1 profile in CVID patients with non-infectious clinical complications. *Clin. Immunol.* 197:130–138. <https://doi.org/10.1016/j.clim.2018.09.006>
23. Urschel, S., L. Kayikci, U. Wintergerst, G. Notheis, A. Jansson, and B.H. Belohradsky. 2009. Common variable immunodeficiency disorders in children: Delayed diagnosis despite typical clinical presentation. *J. Pediatr.* 154:888–894. <https://doi.org/10.1016/j.jpeds.2008.12.020>
24. van de Ven, A.A., L. van de Corput, C.M. van Tilburg, K. Tesselaar, R. van Gent, E.A.M. Sanders, M. Boes, A.C. Bloem, and J.M. van Montfrans. 2010. Lymphocyte characteristics in children with common variable immunodeficiency. *Clin. Immunol.* 135:63–71. [https://doi.org/10.1016/j](https://doi.org/10.1016/j.clim.2009.11.010)[.clim.2009.11.010](https://doi.org/10.1016/j.clim.2009.11.010)
25. Nepesov, S., F.D. Aygun, S. Firtina, H. Cokugras, and Y. Camcioglu. 2020. Clinical and immunological features of 44 common variable im- munodeficiency patients: The experience of a single center in Turkey. *Allergol. Immunopathol.* 48:675–685. [https://doi.org/10.1016/j.aller.2019](https://doi.org/10.1016/j.aller.2019.12.008)[.12.008](https://doi.org/10.1016/j.aller.2019.12.008)
26. Tessarin, G., M. Baronio, L. Gazzurelli, S. Rossi, M. Chiarini, D. Moratto, S.C. Giliani, M.P. Bondioni, R. Badolato, and V. Lougaris. 2023. Ritux- imab monotherapy is effective as first-line treatment for granulomatous lymphocytic interstitial lung disease (GLILD) in CVID patients. *J. Clin.* *Immunol.* 43:2091–2103. <https://doi.org/10.1007/s10875-023-01587-4>
27. Michel, M., V. Chanet, L. Galicier, M. Ruivard, Y. Levy, O. Hermine, E. Oksenhendler, A. Schaeffer, P. Bierling, and B. Godeau. 2004. Autoim- mune thrombocytopenic purpura and common variable immunodefi- ciency: Analysis of 21 cases and review of the literature. *Medicine (Madr).* 83:254–263. <https://doi.org/10.1097/01.md.0000133624.65946.40>
28. Beaton, T.J., D. Gillis, K. Morwood, and M. Bint. 2020. Granulomatous lymphocytic interstitial lung disease: Limiting immunosuppressive therapy-a single-centre experience. *Respirol. Case Rep.* 8:e00565. <https://doi.org/10.1002/rcr2.565>

99. Bride, K.L., T. Vincent, K. Smith-Whitley, M.P. Lambert, J.J. Bleesing, A.E. Seif, C.S. Manno, J. Casper, S.A. Grupp, and D.T. Teachey. 2016. Sirolimus is effective in relapsed/refractory autoimmune cytopenias: Results of a prospective multi-institutional trial. *Blood*. 127:17–28. <https://doi.org/10.1182/blood-2015-07-657981>

1. Chua, I., R. Standish, S. Lear, M. Harbord, E. Eren, M. Raeiszadeh, S. Workman, and D. Webster. 2007. Anti-tumour necrosis factor-alpha therapy for severe enteropathy in patients with common variable im- munodeficiency (CVID). *Clin. Exp. Immunol.* 150:306–311. [https://doi](https://doi.org/10.1111/j.1365-2249.2007.03481.x)[.org/10.1111/j.1365-2249.2007.03481.x](https://doi.org/10.1111/j.1365-2249.2007.03481.x)
2. Deya-Martinez, A., A. Esteve-Sole, N. Velez-Tirado, V. Celis, J. Costa, M. Cols, C. Jou, A. Vlagea, A.M. Plaza-Martin, M. Juan, and L. Alsina. 2018. Sirolimus as an alternative treatment in patients with granulomatous- lymphocytic lung disease and humoral immunodeficiency with im- paired regulatory T cells. *Pediatr. Allergy Immunol.* 29:425–432. [https://](https://doi.org/10.1111/pai.12890)[doi.org/10.1111/pai.12890](https://doi.org/10.1111/pai.12890)
3. von Spee-Mayer, C., C. Echternach, P. Agarwal, S. Gutenberger, V. Soetedjo, S. Goldacker, and K. Warnatz. 2021. Abatacept use is associ- ated with steroid dose reduction and improvement in fatigue and CD4- dysregulation in CVID patients with interstitial lung disease. *J. Allergy Clin. Immunol. Pract.* 9:760–770.e10. [https://doi.org/10.1016/j.jaip.2020](https://doi.org/10.1016/j.jaip.2020.10.028)
4. Rider, N.L., L. Bastarache, J.T. Anderson, E.M. Behrens, N. Chaimowitz, S. Chandrakasan, B. Hartline, A. Liu, R.A. Marsh, and J.A. Connelly. 2025. Expert-based, institutional approaches for reducing the diag- nostic odyssey of patients with inborn errors of immunity. *J. Allergy Clin.* *Immunol. Pract.* 13:1317–1324. <https://doi.org/10.1016/j.jaip.2025.03.040>
5. Matthew Schindler, S.P., Gulbu Uzel, Daniel Reich, and Irene Cortese. 2017. Severe recurrent neuroinflammation associated with cytotoxic T-lymphocyte antigen-4 (CTLA4) haploinsufficiency (P1.330). *Neurol* *ogy*. 88. <https://doi.org/10.1212/WNL.88.16_supplement.P1.330>
6. Schwab, C., A. Gabrysch, P. Olbrich, V. Patiño, K. Warnatz, D. Wolff, A. Hoshino, M. Kobayashi, K. Imai, M. Takagi, et al. 2018. Phenotype, penetrance, and treatment of 133 cytotoxic T-lymphocyte antigen 4- insufficient subjects. *J. Allergy Clin. Immunol.* 142:1932–1946. [https://](https://doi.org/10.1016/j.jaci.2018.02.055) [doi.org/10.1016/j.jaci.2018.02.055](https://doi.org/10.1016/j.jaci.2018.02.055)
7. Tesch, V.K., H. Abolhassani, B. Shadur, J. Zobel, Y. Mareika, S. Shar- apova, E. Karakoc-Aydiner, J.G. Rivière, M. Garcia-Prat, N. Moes, et al. 2020. Long-term outcome of LRBA deficiency in 76 patients after var- ious treatment modalities as evaluated by the immune deficiency and dysregulation activity (IDDA) score. *J. Allergy Clin. Immunol.* 145: 1452–1463. <https://doi.org/10.1016/j.jaci.2019.12.896>
8. Lorenzini, T., M. Fliegauf, N. Klammer, N. Frede, M. Proietti, A. Bula- shevska, N. Camacho-Ordonez, M. Varjosalo, M. Kinnunen, E. de Vries, et al. 2020. Characterization of the clinical and immunologic phenotype and management of 157 individuals with 56 distinct heterozygous NFKB1 mutations. *J. Allergy Clin. Immunol.* 146:901–911. [https://doi.org/](https://doi.org/10.1016/j.jaci.2019.11.051) [10.1016/j.jaci.2019.11.051](https://doi.org/10.1016/j.jaci.2019.11.051)
9. Maccari, M.E., M. Wolkewitz, C. Schwab, T. Lorenzini, J.W. Leiding, N. Aladjdi, H. Abolhassani, W. Abou-Chahla, A. Aiuti, S. Azarnoush, et al. 2023. Activated phosphoinositide 3-kinase δ syndrome: Update from the ESID Registry and comparison with other autoimmune- lymphoproliferative inborn errors of immunity. *J. Allergy Clin. Im-* *munol.* 152:984–996.e10. <https://doi.org/10.1016/j.jaci.2023.06.015>
10. Klemann, C., N. Camacho-Ordonez, L. Yang, Z. Eskandarian, J.L. Rojas- Restrepo, N. Frede, A. Bulashevska, M. Heeg, M.S. Al-Ddafari, J. Premm, et al. 2019. Clinical and Immunological phenotype of patients with primary immunodeficiency due to damaging mutations in NFKB2. *Front. Immunol.* 10:297. <https://doi.org/10.3389/fimmu.2019.00297>
11. Tuijnenburg, P., Allen H. Lango, S.O. Burns, D. Greene, M.H. Jansen, E. Staples, J. Stephens, K.J. Carss, D. Biasci, H. Baxendale, et al. 2018. Loss- of-function nuclear factor kappaB subunit 1 (NFKB1) variants are the most common monogenic cause of common variable immunodeficiency in Europeans. *J. Allergy Clin. Immunol.* 142:1285–1296. [https://doi.org/10](https://doi.org/10.1016/j.jaci.2018.01.039)[.1016/j.jaci.2018.01.039](https://doi.org/10.1016/j.jaci.2018.01.039)
12. Bez, P., B. Smits, C. Geier, A. Hirsch, A. Caballero de Oyteza, M. Proietti, B. Grimbacher, M. Wolkewitz, S. Goldacker, and K. Warnatz. 2025. Uncovering risk factors of premature mortality in common variable immunodeficiency. *J. Allergy Clin. Immunol. Pract.* 13:1201–1209.e10. <https://doi.org/10.1016/j.jaip.2025.03.009>
13. Rojas-Restrepo, J., A. Caballero-Oteyza, K. Huebscher, H. Haberstroh, M. Fliegauf, B. Keller, R. Kobbe, K. Warnatz, S. Ehl, M. Proietti, and B. Grimbacher. 2021. Establishing the molecular diagnoses in a cohort of 291 patients with predominantly antibody deficiency by targeted next-generation sequencing: Experience from a monocentric study. *Front.* *Immunol.* 12:786516. <https://doi.org/10.3389/fimmu.2021.786516>
14. Hillier, K., G. Yuen, A. Hui, S. Kumar, P.G. Roy, J.T. McColgan, K. Zal- dana, H. Allard-Chamard, K. Premo, N. Kaneko, et al. 2025. Loss of B cell tolerance at the T2/T3a B cell transition is a convergent pathogenic mechanism in common variable immunodeficiency. *bioRxiv*. [https://doi](https://doi.org/10.1101/2025.06.07.658167)

[.org/10.1101/2025.06.07.658167](https://doi.org/10.1101/2025.06.07.658167) (Preprint posted June 11, 2025).

1. Fang, M., D. Wang, and E. Selvin. 2024. Prevalence of Type 1 diabetes among US children and adults by age, sex, race, and ethnicity. *JAMA*. 331:1411–1413. <https://doi.org/10.1001/jama.2024.2103>
2. Wyne, K.L., L. Nair, C.P. Schneiderman, B. Pinsky, O. Antunez Flores, D. Guo, B. Barger, and A.H. Tessnow. 2022. Hypothyroidism prevalence in the United States: A retrospective study combining national health and nutrition examination survey and claims data, 2009-2019. *J. Endocr. Soc.* 7:bvac172. <https://doi.org/10.1210/jendso/bvac172>
3. CDC. 2023. Most recent national asthma data. [https://www.cdc.gov/](https://www.cdc.gov/asthma/most_recent_national_asthma_data.htm) [asthma/most_recent_national_asthma_data.htm](https://www.cdc.gov/asthma/most_recent_national_asthma_data.htm) (accessed June 25, 2025).
4. Correa-Jimenez, O., S. Restrepo-Gualteros, G. Nino, C. Cunningham- Rundles, K.E. Sullivan, R.L. Fuleihan, and M.J. Gutierrez. 2023. Respi- ratory comorbidities associated with bronchiectasis in patients with common variable immunodeficiency in the USIDNET registry. *J. Clin.* *Immunol.* 43:2208–2220. <https://doi.org/10.1007/s10875-023-01593-6>
5. Aaron, S.D., K.L. Vandemheen, J.M. FitzGerald, M. Ainslie, S. Gupta, C. Lemière, S.K. Field, R.A. McIvor, P. Hernandez, I. Mayers, et al. 2017.

Reevaluation of diagnosis in adults with physician-diagnosed asthma. *JAMA*. 317:269–279. <https://doi.org/10.1001/jama.2016.19627>

1. Janssen, L.M.A., M. van der Flier, and E. de Vries. 2021. Lessons learned from the clinical presentation of common variable immunodeficiency disorders: A systematic review and meta-analysis. *Front. Immunol.* 12: 620709. <https://doi.org/10.3389/fimmu.2021.620709>
2. Mucke, J., A. Cornet, T. Witte, and M. Schneider. 2022. Association of common variable immunodeficiency and rare and complex connective tissue and musculoskeletal diseases. A systematic literature review. *Clin. Exp. Rheumatol.* 40:40–45. [https://doi.org/10.55563/](https://doi.org/10.55563/clinexprheumatol/bbuvih)[clinexprheumatol/bbuvih](https://doi.org/10.55563/clinexprheumatol/bbuvih)
3. Szczawin´ska-Popłonyk, A., J. Bekalarska, K. Je˛ch, N. Knobloch, O. Łukasik, A. Ossowska, J. Ruducha, and Z. Wysocka. 2025. The burden of non-infectious organ-specific immunopathology in pediatric common variable immunodeficiency. *Int. J. Mol. Sci.* 26:2653. [https://doi.org/10](https://doi.org/10.3390/ijms26062653)[.3390/ijms26062653](https://doi.org/10.3390/ijms26062653)
4. Page, M.J., J.E. McKenzie, P.M. Bossuyt, I. Boutron, T.C. Hoffmann, C.D. Mulrow, L. Shamseer, J.M. Tetzlaff, E.A. Akl, S.E. Brennan, et al. 2021. The PRISMA 2020 statement: An updated guideline for reporting sys- tematic reviews. *BMJ*. 372:n71. <https://doi.org/10.1136/bmj.n71>
5. Cumpston, M., T. Li, M.J. Page, J. Chandler, V.A. Welch, J.P. Higgins, and J. Thomas. 2019. Updated guidance for trusted systematic reviews: A new edition of the Cochrane handbook for systematic reviews of in- terventions. *Cochrane Database Syst. Rev.* 10:ED000142. [https://doi.org/](https://doi.org/10.1002/14651858.ED000142) [10.1002/14651858.ED000142](https://doi.org/10.1002/14651858.ED000142)
