# Supplementary material for: A systematic literature review of CVID reveals pervasive detrimental noninfectious manifestations
Source: J Hum Immun. 2025 Oct 24;2(1):e20250157. doi: 10.70962/jhi.20250157 (PMC13177467; doi:10.70962/jhi.20250157)
Supplement: Table S5 — shows search strategy. [file jhi_20250157_tables5.docx]

Table S5: Search strategy

| No. | Strings | Hits |
| --- | --- | --- |
| Search strategy for Embase | | |
| 1 | Clinical study/ | 166,410 |
| 2 | Case control study/ | 215,751 |
| 3 | Family study/ | 25,812 |
| 4 | Longitudinal study/ | 210,681 |
| 5 | Retrospective study/ | 1,600,186 |
| 6 | Prospective study/ | 913,467 |
| 7 | Randomized controlled trials/ | 272,233 |
| 8 | 6 not 7 | 902,341 |
| 9 | Cohort analysis/ | 1,146,341 |
| 10 | (Cohort adj (study or studies)).mp. | 509,883 |
| 11 | (Case control adj (study or studies)).tw. | 174,809 |
| 12 | (follow up adj (study or studies)).tw. | 75,785 |
| 13 | (observational adj (study or studies)).tw. | 273,136 |
| 14 | (epidemiologic$ adj (study or studies)).tw. | 125,278 |
| 15 | (cross sectional adj (study or studies)).tw. | 368,564 |
| 16 | or/1-5,8-15 | 4,188,560 |
| 17 | Clinical Trial/ | 1,080,867 |
| 18 | trial.ti,ab. | 1,167,269 |
| 19 | Randomized Controlled Trial/ | 816,540 |
| 20 | controlled clinical trial/ | 472,861 |
| 21 | multicenter study/ | 389,531 |
| 22 | Phase 3 clinical trial/ | 74,930 |
| 23 | Phase 4 clinical trial/ | 7,087 |
| 24 | exp RANDOMIZATION/ | 99,509 |
| 25 | Single Blind Procedure/ | 54,293 |
| 26 | Double Blind Procedure/ | 217,870 |
| 27 | Crossover Procedure/ | 77,632 |
| 28 | PLACEBO/ | 411,424 |
| 29 | randomi?ed controlled trial$.tw. | 343,105 |
| 30 | rct.tw. | 57,236 |
| 31 | (random$ adj2 allocat$).tw. | 57,040 |
| 32 | single blind$.tw. | 32,956 |
| 33 | double blind$.tw. | 251,028 |
| 34 | ((treble or triple) adj blind$).tw. | 2,076 |
| 35 | placebo$.tw. | 377,645 |
| 36 | Prospective Study/ | 913,467 |
| 37 | or/17-36 | 3,453,266 |
| 38 | abstract report/ | 89,835 |
| 39 | Conference proceeding.pt. | - |
| 40 | Conference abstract.pt. | 5,107,145 |
| 41 | Editorial.pt. | 802,165 |
| 42 | or/38-41 | 5,998,868 |
| 43 | 37 not 42 | 2,566,265 |
| 44 | exp cohort analysis/ | 1,146,341 |
| 45 | exp longitudinal study/ | 210,900 |
| 46 | exp prospective study/ | 913,467 |
| 47 | exp follow up/ | 2,172,864 |
| 48 | cohort$.tw. | 1,561,413 |
| 49 | exp case control study/ | 234,221 |
| 50 | (case$ and control$).tw. | 923,384 |
| 51 | (case$ and series).tw. | 348,569 |
| 52 | or/44-51 | 5,157,370 |
| 53 | 16 or 43 or 52 | 8,056,926 |
| 54 | Conference proceeding.pt. | - |
| 55 | Conference abstract.pt. | 5,107,145 |
| 56 | 54 or 55 | 5,107,145 |
| 57 | 53 not 56 | 6,179,562 |
| 58 | 57 and (common variable immunodeficiency/ or (common variable immunodeficiency or common variable immune deficiency or CVID).ti,ab.) | 1,427 |
| 59 | limit 58 to english language | 1,358 |
| Search strategy for MEDLINE | | |
| 1 | exp cohort studies/ | 2,593,433 |
| 2 | cohort$.tw. | 929,953 |
| 3 | controlled clinical trial.pt. | 95,511 |
| 4 | epidemiologic methods/ | 31,618 |
| 5 | limit 4 to yr=1966-1989 | 11,199 |
| 6 | exp case-control studies/ | 1,496,742 |
| 7 | (case$ and control$).tw. | 630,314 |
| 8 | (case$ and series).tw. | 242,701 |
| 9 | or/1-3,5-8 | 3,856,539 |
| 10 | Randomized Controlled Trials as Topic/ | 168,942 |
| 11 | randomized controlled trial/ | 610,719 |
| 12 | Random Allocation/ | 107,079 |
| 13 | Double Blind Method/ | 178,009 |
| 14 | Single Blind Method/ | 33,371 |
| 15 | clinical trial/ | 539,706 |
| 16 | clinical trial, phase i.pt. | 25,833 |
| 17 | clinical trial, phase ii.pt. | 41,080 |
| 18 | clinical trial, phase iii.pt. | 22,566 |
| 19 | clinical trial, phase iv.pt. | 2,484 |
| 20 | controlled clinical trial.pt. | 95,511 |
| 21 | randomized controlled trial.pt. | 610,719 |
| 22 | multicenter study.pt. | 344,479 |
| 23 | clinical trial.pt. | 539,706 |
| 24 | exp Clinical Trials as topic/ | 390,675 |
| 25 | or/10-24 | 1,601,306 |
| 26 | (clinical adj trial$).tw. | 508,408 |
| 27 | ((singl$ or doubl$ or treb$ or tripl$) adj (blind$3 or mask$3)).tw. | 204,305 |
| 28 | PLACEBOS/ | 35,935 |
| 29 | placebo$.tw. | 255,310 |
| 30 | randomly allocated.tw. | 38,364 |
| 31 | (allocated adj2 random$).tw. | 42,270 |
| 32 | or/26-31 | 821,783 |
| 33 | 25 or 32 | 1,973,696 |
| 34 | case study/ | 2,396,137 |
| 35 | case report.tw. | 422,932 |
| 36 | historical article/ | 369,373 |
| 37 | or/34-36 | 2,825,028 |
| 38 | 33 not 37 | 1,954,740 |
| 39 | Epidemiologic studies/ | 9,522 |
| 40 | exp case control studies/ | 1,496,742 |
| 41 | exp cohort studies/ | 2,593,433 |
| 42 | Case control.tw. | 161,233 |
| 43 | (cohort adj (study or studies)).tw. | 347,407 |
| 44 | Cohort analy$.tw. | 12,907 |
| 45 | (Follow up adj (study or studies)).tw. | 57,970 |
| 46 | (observational adj (study or studies)).tw. | 176,225 |
| 47 | Longitudinal.tw. | 342,873 |
| 48 | Retrospective.tw. | 804,152 |
| 49 | Cross sectional.tw. | 556,579 |
| 50 | Cross-sectional studies/ | 498,587 |
| 51 | or/39-50 | 3,976,887 |
| 52 | 9 or 38 or 51 | 6,052,065 |
| 53 | 52 and (common variable immunodeficiency/ or (common variable immunodeficiency or common variable immune deficiency or CVID).ti,ab.) | 1,006 |
| 54 | limit 53 to english language | 938 |
| Search strategy for Cochrane Register of Controlled Trials | | |
| 1 | common variable immunodeficiency/ or (common variable immunodeficiency or common variable immune deficiency or CVID).ti,ab. | 67 |
| 2 | limit 1 to english language | 65 |

Embase 1974 to 2024 April 12; Executed: April 15, 2024

Ovid MEDLINE(R) and Epub Ahead of Print, In-Process, In-Data-Review & Other Non-Indexed Citations, Daily and Versions 1946 to April 12, 2024; Executed: April 15, 2024

EBM Reviews - Cochrane Central Register of Controlled Trials March 2024; Executed: April 15, 2024

*EBM, Evidence-Based Medicine; Embase, Excerpta Medica Database; MEDLINE, Medical Literature Analysis and Retrieval System Online.*
